# Outcome measures in facial prosthesis research: A systematic review

**DOI:** 10.1016/j.prosdent.2020.09.010

**Published:** 2021-12

**Authors:** Rachael Y. Jablonski, Benjamin J. Veale, Trevor J. Coward, Andrew J. Keeling, Chris Bojke, Sue H. Pavitt, Brian R. Nattress

**Affiliations:** a Specialty Registrar in Restorative Dentistry and NIHR Doctoral Fellow, Department of Restorative Dentistry, School of Dentistry, University of Leeds, Leeds, UK; b Medical Student, Hull York Medical School, University of York, York, UK; c Reader and Honorary Consultant in Maxillofacial and Craniofacial Rehabilitation, Academic Centre of Reconstructive Science, Faculty of Dentistry, Oral and Craniofacial Sciences, King’s College London, London, UK; d Clinical Associate Professor, Department of Restorative Dentistry, School of Dentistry, University of Leeds, Leeds, UK; e Professor of Health Economics, Academic Unit of Health Economics, School of Medicine, University of Leeds, Leeds, UK; f Professor of Translational and Applied Health Research, Dental Translational and Clinical Research Unit, School of Dentistry, University of Leeds, Leeds, UK; g Clinical Professor and Honorary Consultant, Department of Restorative Dentistry, School of Dentistry, University of Leeds, Leeds, UK

## Abstract

**Statement of problem:**

Facial prosthesis research uses a wide variety of outcome measures, which results in challenges when comparing the effectiveness of interventions among studies. Consensus is lacking regarding the most appropriate and meaningful outcome measures to use in facial prosthesis research to capture important perspectives.

**Purpose:**

The purpose of the systematic review was to identify and synthesize outcome measures used in facial prosthesis research.

**Material and methods:**

Electronic searches were performed in 11 databases (including nonpeer-reviewed literature). The citations were searched, and expert societies were contacted to identify additional studies. Inclusion criteria comprised studies of participants with facial defects who required or had received prosthetic rehabilitation with an external facial prosthesis. Exclusion criteria comprised participants with ocular prostheses, case reports, case series with fewer than 5 participants, laboratory-based studies, and studies published before 1980. Study selection was performed independently by 2 reviewers. Discrepancies were resolved through discussion or by a third reviewer. Outcome measures were synthesized with a categorization approach based on the perspective, theme, and subtheme of the outcome measures. Quality assessment was performed with an appraisal tool that enabled evaluation of studies with diverse designs.

**Results:**

Database searching identified 13 058 records, and 7406 remained after duplications were removed. After initial screening, 189 potentially relevant records remained, and 186 full texts were located (98% retrieval rate). After full-text screening, 124 records were excluded. Citation searches and contact with expert societies identified 4 further records. In total, 69 articles (grouped into 65 studies) were included. Studies were categorized as per the perspective of their outcome measures, with the following findings: patient-reported (74% of studies), clinical indicators (34%), clinician-reported (8%), multiple viewpoints (6%), and independent observer-reported (3%). Patient-reported outcome measures included tools to assess satisfaction, quality of life, and psychologic health. Variability in the choice of outcome measures was evident among the studies, with many self-designed, unvalidated, condition-specific questionnaires reported. A greater number of outcome measure themes emerged over time; themes such as service delivery and health state utility have recently been evaluated.

**Conclusions:**

Over the past 40 years, facial prosthesis research has focused on patient-reported outcome measures. Outcome measures relating to other perspectives have been used less frequently, although new themes appear to be emerging in the literature. Future research should use outcome measures with appropriate measurement properties for use with facial prosthetics.

**Clinical Implications**

The wide variety of outcome measures used in facial prosthesis research highlights the need for validated, standardized outcome measures that capture a range of perspectives. Evidence-based approaches that use validated, condition-specific, patient-reported outcome measures allow for systematic comparison and comprehensive evaluation of facial prosthetic rehabilitation and its benefit to patients. More systematic protocols of assessment are required to capture outcomes from the perspective of the clinician, independent observer, or multiple viewpoints.

Facial defects may result from congenital or acquired conditions^1^ and can result in multiple psychosocial and functional impairments.^2^ The 2 main approaches to rehabilitating patients with facial defects are surgical reconstruction or prosthetic rehabilitation.^1^ Surgical reconstruction can provide a long-term solution to replacing the missing tissue. However, it may be unsuitable depending on the extent of tissue loss, the availability of donor tissue, the patient’s psychophysical condition, and technical challenges.^1^^,^^3^

Removable facial prostheses can provide an esthetic and functional outcome without the associated risks of reconstructive surgery.^1^ Studies have evaluated the impact of facial prostheses on quality of life (QoL),^4^, ^5^, ^6^, ^7^, ^8^, ^9^ psychologic health,^4^ and satisfaction.^2^^,^^10^ From a service delivery perspective, the conventional manufacture of facial prostheses is regarded as time consuming, labor intensive, and technically challenging.^11^^,^^12^ The ongoing impact on patients and healthcare services is evident with the need for regular maintenance and replacement.^12^

A variety of innovations in facial prosthesis rehabilitation have occurred in recent decades. In the late 1970s, osseointegrated implants were introduced to overcome some of the limitations of conventional retention methods.^13^ From the late 1990s, digital technology has been introduced to supplement or replace steps in conventional manufacturing,^14^ as summarized in a recent systematic review.^15^

Clinical management of patients with facial defects should adopt an evidence-based approach. Facial prosthesis research uses a wide variety of outcome measures, which results in challenges when comparing the effectiveness of interventions among studies. In addition, a consensus is lacking regarding the most appropriate and meaningful outcome measures to use in facial prosthesis research to capture important perspectives and outcomes.

The purpose of this systematic review was to identify and synthesize outcome measures used in facial prosthesis research. The scope of the review was purposefully broad to map the outcome measures used over time. Quality assessment was planned to provide a holistic overview of the quality of studies and to identify broad areas where reporting was lacking. Anticipating a heterogeneous group of studies, the Quality Assessment Tool for Studies of Diverse Designs (QATSDD) was selected.^16^ To the best of the authors’ knowledge, a similar systematic review had not been undertaken previously or registered on prospective databases.

## Material and methods

The systematic review was based on established guidance.^17^ Recently published systematic reviews that synthesized outcome measures or outcomes from the dental literature were also consulted.^18^^,^^19^ The protocol was registered in an international prospective register of systematic reviews.^20^ The review question was “What outcome measures are used to capture the outcomes of facial prosthesis provision in patients with facial defects requiring prosthetic rehabilitation?”

Table 1 summarizes the eligibility criteria. The population of interest was participants with facial defects who required or had received an external facial prosthesis. Studies of ocular prostheses were excluded because of anticipated differences in treatment delivery and evaluation. There were no age restrictions, and facial defects of any underlying etiology, extent, and recency were included. Differences in these factors were considered as sources of clinical diversity and potential reasons for variability in the outcome measures used. Studies published over the last 40 years (January 1980 to 2020) were included as a comprehensive overview. This time period might also identify trends commensurate with changes in retention and manufacturing methods.^13^^,^^15^

**Table 1:** Inclusion and exclusion criteria

Category	Included	Excluded
Population	Participants with facial defects who required or had received prosthetic rehabilitation. Facial defects of any underlying etiology, extent, or recency. No age restrictions.	Participants with facial defects not requiring prosthetic rehabilitation. Studies of participants with ocular prostheses only.
Intervention	Studies of facial prostheses with any retention method, manufacturing technique, or materials.	—
Comparator	For comparative studies, any treatment for facial defects, no treatment, or unaffected comparator group.	—
Outcomes	Any evaluation of facial prosthesis provision. Any adverse effects.	Prevalence or etiology of facial defects.
Study type	Systematic reviews with meta-analysis. Experimental studies. Observational studies. Health economics studies. Mixed methods studies.	Nonsystematic literature reviews and systematic reviews without meta-analysis. Case reports, case studies, and case series with fewer than 5 participants. Conference abstracts with inadequate information regarding methodology or outcome measures. Laboratory based in vitro studies. Letters.
Characteristics	Studies originating from any country.	Studies published before 1980. Studies not available in full-text English after reasonable attempts to obtain.

Electronic searches were performed in EMBASE, MEDLINE, PsycINFO, Web of Science Core Collection, Cochrane Library, and CINAHL from inception to the present day. Nonpeer-reviewed literature databases were searched to minimize publication bias by using the International Clinical Trials Registry Platform, Clinicaltrials.gov, Opengrey, ProQuest Dissertation and Theses A&I, and Networked Digital Library of Theses and Dissertations. Reference lists of included articles were manually searched, and citations were searched in Scopus. In addition, 2 societies (American Academy of Maxillofacial Prosthetics and Institute of Maxillofacial Prosthetists Technologists) were contacted through e-mail to identify missing or unpublished studies.

The search strategy was developed and tailored to each database with support from an information specialist. The searches were performed in November 2019 and comprised a combination of Medical Subject Headings and free text keywords. One main concept was searched relating to the intervention. No further concepts were used as a population concept would overlap with the intervention concept. Furthermore, a concept relating to the outcomes was not used, as outcomes are often not well described in abstracts or well indexed with controlled vocabulary terms.^21^ There were no language or time restrictions. It was anticipated that this would result in a highly sensitive but less precise search. Where possible, limits and filters were applied to exclude letters and in vitro studies. All searches were documented in a search log, and the search strategy for EMBASE is included in Table 2.

**Table 2:** EMBASE search strategy

Database: Embase Classic+Embase <1947 to 2019 November 07>	
1	exp facial prosthesis/(14)
2	∗maxillofacial prosthesis/or ∗ear prosthesis/or ∗nose prosthesis/(1225)
3	exp ∗artificial eye/(168)
4	((maxillo?facial or cranio?facial or extra?oral or face or facial or orbit or orbital or ocular or eye or eyes or auricular or ear or ears or nasal or nose? or cheek?) adj2 prosth∗).tw. (2704)
5	((maxillo?facial or cranio?facial or extra?oral or face or facial or orbit or orbital or ocular or eye or eyes or auricular or ear or ears or nasal or nose? or cheek?) adj2 epithes∗).tw. (58)
6	((maxillo?facial or cranio?facial or extra?oral or face or facial or orbit or orbital or ocular or eye or eyes or auricular or ear or ears or nasal or nose? or cheek?) adj1 artificial).tw. (761)
7	or/1-6 (4099)
8	exp animals/not exp humans/(5330008)
9	exp nonhuman/not exp human/(4497883)
10	exp experimental animal/(680503)
11	exp veterinary medicine/(45929)
12	animal experiment/(2452244)
13	or/8-12 (7533946)
14	7 not 13 (3943)
15	limit 14 to letter (32)
16	14 not 15 (3911)

The studies were imported into a reference management software program (EndNote X8; Clarivate Analytics). Duplicates were removed with the software program, and a sample of studies was checked manually to ensure the process was reliable. Screening of titles and abstracts was undertaken independently by 2 reviewers (R.J., B.V.), and the full text of any potentially relevant reports were retrieved. Two reviewers (R.J., B.V.) independently screened the full-text articles for compliance with the inclusion and exclusion criteria. The criteria were initially piloted on sample reports to ensure they could be applied consistently. Any discrepancies were resolved through consensus or by consulting an additional reviewer (C.B., S.P., B.N.). All potentially relevant articles excluded from the review were listed in a table of the characteristics of excluded studies.

A tailored data extraction form was created, piloted, and developed based on available checklists.^22^ Data were extracted from included studies by 1 reviewer (R.J.) and checked for accuracy by a second reviewer (B.V.). Multiple reports of the same study were linked, and data were collected on a single form. The following items were extracted: author details, publication year, country, design, participant characteristics, participant numbers, intervention, comparator, adverse outcomes, and outcome measures. A descriptive approach was used to categorize study design as some studies did not fit discretely with explicit study design definitions and there was variable quality of reporting. Outcome measures were not extracted if they related to concepts other than the facial prosthesis itself (such as those relating to bone-anchored hearing aids or peri-implantitis). Attempts were made to contact study authors to obtain missing data.

A diverse range of study designs was anticipated, and therefore, 2 appraisal approaches were possible. First, the different study designs could be separated and evaluated with multiple appraisal tools specific to each study type.^16^ Second, all study types could be appraised with a standardized, pragmatic approach with a generic quality assessment tool such as the QATSDD.^16^ The second approach was in keeping with the purpose of the systematic review.

Preliminary assessments of the quality assessment tool for studies with diverse designs (QATSDD) indicate its usefulness to standardize quality assessment approaches when dealing with diverse study designs.^16^ It enabled a pragmatic, holistic evaluation of the overall body of evidence and allowed broad quality comparisons to be drawn among different study types.^16^ The main limitations related to the broad nature of the tool, which may not be appropriate for all types of research.^16^ It was also not designed to replace quality assessment tools for specific approaches (for example, systematic reviews based entirely on randomized controlled trials).^16^ The tool has been assessed in the disciplines of psychology, sociology, and nursing^16^ and has recently been used to assess dental studies.^23^, ^24^, ^25^

The QATSDD tool has a total of 16 criteria; of which, 14 apply to qualitative studies, 14 apply to quantitative studies, and all 16 apply to mixed methods research.^16^ During quality assessment, each study was awarded a score on a scale of 0 to 3 for all relevant criteria.^16^ A score of 3 was awarded when a criterion was completely met. Some criteria lacked clarity, which led to some statements being interpreted differently by the reviewers.^26^ Therefore, 2 reviewers (R.J., B.V.) agreed on what would be expected of studies for each statement to ensure consistency of application. Any disagreements were resolved through an iterative process.^16^ Each study was given an overall quality score, expressed as a percentage of the maximum possible score.^16^ While the tool is useful to direct dialog and provide a general overview of study quality, overall quality scores should be interpreted with caution because of the equal weighting of all criteria.^24^^,^^26^

A list of outcome measures was compiled and synthesized based on a categorization approach.^18^ Category names were agreed based on the perspective of the evaluator.^18^ Five categories were developed, including patient-reported outcome measures (PROMs), clinician-reported outcome measures, independent observer-reported outcome measures, outcome measures encompassing multiple perspectives, and clinical indicators. Themes and subthemes were used to subcategorize outcome measures based on the concepts evaluated. For example, the PROM category was subdivided into themes relating to satisfaction, QoL, psychologic health, and other concepts. The QoL theme was then divided into subthemes such as condition specific (relating to facial prostheses), condition specific (not relating to facial prostheses), and generic tools.

## Results

**Figure 1:**
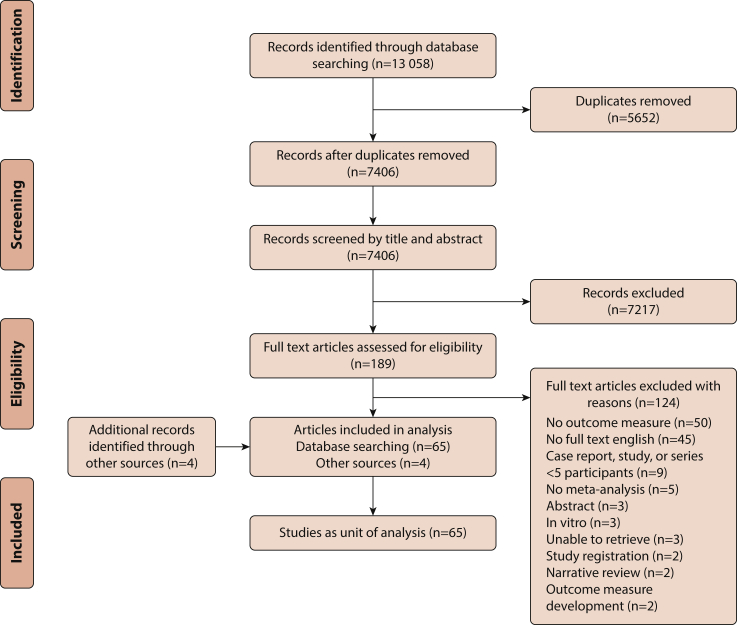
PRISMA flow diagram of study selection process. PRISMA, preferred reporting items for systematic reviews and meta-analyses.

From the database searches, 13 058 records were identified, and 7406 records remained after the removal of duplicates. After screening titles and abstracts, 189 potentially relevant records remained, and 186 full texts were located (98% retrieval rate). After screening full texts, 124 records were excluded principally because of lack of an explicit outcome measure related to facial prostheses (n=50) or lack of availability of a full-text English manuscript (n=45). Citation searches and contact with expert societies identified 4 further records. In total, 69 full-text articles were included, which were grouped into 65 studies (Fig. 1).

Characteristics of included studies are outlined in Supplemental Tables 1-5^27^, ^28^, ^29^, ^30^ (available online). Study designs included 2 experimental studies (3%), 14 cross-sectional studies (22%), 14 prospective longitudinal observational studies (22%), 33 retrospective longitudinal observational studies (51%), and 2 mixed-methods studies (3%). Most studies originated in Europe (55%). Studies focused on adults (≥18 years) in 25 studies (38%), children in 3 studies (5%), both age groups in 31 studies (48%), and 6 studies were not explicit (9%). Different types of facial defect were evaluated in 29 studies (45%), whereas 22 studies evaluated auricular defects only (34%), 7 studies evaluated orbital defects (11%), 5 studies evaluated nasal defects (8%), and 2 studies were not explicit (3%). The underlying etiology included oncology or resections in 13 studies (20%), congenital conditions in 4 studies (6%), diverse etiologies in 43 studies (66%), and 5 studies were not explicit (8%).

The quality of included studies was assessed with the QATSDD (Supplemental Table 6 [available online]).^16^ Average-quality scores were calculated for the study design groups (Table 3). These comprised experimental studies (61.9%), cross-sectional studies (45.2%), prospective longitudinal observational studies (39.5%), retrospective longitudinal observational studies (41.6%), and mixed-methods studies (37.5%). The broad range of quality scores for the cross-sectional and longitudinal observational studies highlights variability in their quality. Some criteria had consistently low scores among the different groups, including evidence that sample size was considered in terms of analysis, statistical assessment of reliability and validity of measurement tools, and evidence of user involvement in design.

**Table 3:** Average score for each quality criteria in QATSDD tool^16^

QATSDD Criteria	Average (Minimum, Maximum) Score for Each Study Type				
	Experimental (n=2)	Cross-Sectional (n=14)	Prospective Longitudinal (n=14)	Retrospective Longitudinal (n=33)	Mixed Methods (n=2)
Explicit theoretical framework	3.0 (3, 3)	1.8 (1, 3)	1.3 (0, 3)	1.8 (0, 3)	2.0 (2, 2)
Statement of aims/objectives in main body of report	2.0 (2, 2)	1.9 (1, 3)	1.6 (0, 3)	1.7 (1, 2)	1.5 (1, 2)
Clear description of research setting	2.0 (1, 3)	1.7 (1, 3)	1.4 (0, 3)	2.1 (1, 3)	1.0 (1, 1)
Evidence of sample size considered in terms of analysis	1.0 (0, 2)	0.4 (0, 3)	0.2 (0, 1)	0.0 (0, 0)	0.5 (0, 1)
Representative sample of target group of a reasonable size	1.5 (1, 2)	1.9 (1, 2)	1.7 (1, 2)	1.7 (1, 3)	1.5 (1, 2)
Description of procedure for data collection	2.5 (2, 3)	2.0 (1, 3)	2.1 (1, 3)	1.9 (1, 3)	1.5 (1, 2)
Rationale for choice of data collection tool(s)	1.0 (1, 1)	1.4 (0, 3)	1.2 (0, 3)	1.0 (0, 3)	1.0 (1, 1)
Detailed recruitment data	2.0 (1, 3)	2.0 (1, 3)	1.6 (0, 3)	2.1 (1, 3)	2.0 (2, 2)
Statistical assessment of reliability and validity of measurement tool (quantitative)	1.0 (0, 2)	0.6 (0, 3)	0.4 (0, 2)	0.2 (0, 2)	0.5 (0, 1)
Fit between stated research question and method of data collection (quantitative)	2.5 (2, 3)	1.7 (1, 2)	1.6 (0, 3)	1.7 (1, 3)	1.5 (1, 2)
Fit between stated research question and format and content of data collection tool (qualitative)	N/A	N/A	N/A	N/A	1.5 (1, 2)
Fit between research question and method of analysis	2.5 (2, 3)	1.8 (1, 3)	1.4 (0, 3)	1.5 (1, 3)	1.5 (1, 2)
Good justification for analytic method selected	2.5 (2, 3)	0.6 (0, 2)	0.9 (0, 2)	0.6 (0, 2)	0.5 (0, 1)
Assessment of reliability of analytical process (qualitative)	N/A	N/A	N/A	N/A	0.5 (0, 1)
Evidence of user involvement in design	0.5 (0, 1)	0.2 (0, 2)	0.1 (0, 2)	0.0 (0, 0)	0.0 (0, 0)
Strengths and limitations critically discussed	2.0 (2, 2)	1.1 (0, 2)	0.9 (0, 2)	1.1 (0, 2)	1.0 (1, 1)
Overall quality score (as a percentage of total possible score (%))	61.9 (45, 79)	45.2 (26, 76)	39.5 (12, 67)	41.6 (21, 62)	37.5 (35, 40)

N/A, not applicable; QATSDD, quality assessment tool for studies with diverse designs.

Possible score for each criterion in QATSDD tool ranges from 0 to 3.

A total of 117 outcome measures that related to facial prostheses were identified from the 65 studies. Studies were categorized based on perspective, theme, and subtheme of the outcome measures (Table 4). PROMs was the most popular category identified in 48 studies (74%). Within this category, 31 studies evaluated satisfaction, 14 studies evaluated QoL, 6 studies evaluated psychologic health, and 6 studies evaluated other patient-reported outcomes. Table 5 lists the outcome measures that fall within each category and theme. Satisfaction was frequently captured with self-designed condition-specific questionnaires. QoL was assessed through generic and condition-specific tools, including those usually used in other contexts such as plastic surgery or otolaryngology. A broad range of tools to capture psychologic health was also identified.

**Table 4:** Categorization of research studies based on outcome measures

Perspective (Number of Studies)	Theme (Number of Studies)	Subtheme (Number of Studies)
Patient reported (n=48)^2^, ^4^, ^5^, ^6^, ^7^, ^8^, ^9^, ^10^, ^31^, ^32^, ^33^, ^34^, ^35^, ^36^, ^37^, ^38^, ^39^, ^40^, ^41^, ^42^, ^43^, ^44^, ^45^, ^46^, ^47^, ^48^, ^49^, ^50^, ^51^, ^52^, ^53^, ^54^, ^55^, ^56^, ^57^, ^58^, ^59^, ^60^, ^61^, ^62^, ^63^, ^64^, ^65^, ^66^, ^67^, ^68^, ^69^, ^70^	Satisfaction (n=31)^2^^,^^5^^,^^10^^,^^31^, ^32^, ^33^, ^34^, ^35^, ^36^, ^37^, ^38^, ^39^, ^40^, ^41^, ^42^, ^43^, ^44^, ^45^, ^46^, ^47^, ^48^, ^49^, ^50^, ^51^, ^52^, ^53^, ^54^, ^55^, ^56^, ^57^, ^58^	Not applicable
	Quality of life (n=14)^4^, ^5^, ^6^, ^7^, ^8^, ^9^^,^^32^^,^^39^^,^^59^, ^60^, ^61^, ^62^, ^63^, ^64^	Condition specific (specific to facial prostheses) (n=6)^4^, ^5^, ^6^, ^7^, ^8^, ^9^
		Condition specific (not specific to facial prostheses) (n=5)^39^^,^^59^, ^60^, ^61^, ^62^
		Generic (n=7)^4^^,^^5^^,^^32^^,^^39^^,^^59^^,^^63^^,^^64^
	Psychologic health (n=6)^4^^,^^32^^,^^65^, ^66^, ^67^, ^68^	Psychologic health (n=6)^4^^,^^32^^,^^65^, ^66^, ^67^, ^68^
		Psychosocial (n=1)^67^
	Other patient reported (n=6)^32^^,^^41^^,^^44^^,^^61^^,^^69^^,^^70^	Preference (n=1)^44^
		Appearance/function (n=1)^61^
		Function (n=1)^69^
		Functional comfort (n=1)^32^
		Duration of wear (n=1)^70^
		Ability to wear prostheses as desired(n=1)^41^
Clinician reported (n=5)^4^^,^^5^^,^^71^, ^72^, ^73^	Clinical evaluation (n=5)^4^^,^^5^^,^^71^, ^72^, ^73^	Not applicable
Independent observer (n=2)^74^^,^^75^	Utility (n=1)^74^	Not applicable
	Appearance (n=1)^75^	Not applicable
Multiple perspectives (n=4)^66^^,^^76^, ^77^, ^78^	Appearance (n=3)^66^^,^^76^^,^^77^	Not applicable
	Success (n=1)^78^	Not applicable
Clinical indicators (n=22)^8^^,^^33^^,^^39^^,^^44^^,^^47^^,^^54^^,^^57^^,^^58^^,^^65^^,^^69^^,^^70^^,^^72^^,^^73^^,^^79^, ^80^, ^81^, ^82^, ^83^, ^84^, ^85^, ^86^, ^87^	Prosthesis survival (n=10)^33^^,^^39^^,^^57^^,^^73^^,^^79^, ^80^, ^81^, ^82^, ^83^, ^84^	Prosthesis survival/lifespan (n=9)^33^^,^^39^^,^^57^^,^^73^^,^^79^, ^80^, ^81^, ^82^, ^83^
		Prosthesis failure (n=1)^84^
	Aftercare (n=5)^8^^,^^33^^,^^44^^,^^79^^,^^80^	Not applicable
	Complications (n=9)^47^^,^^58^^,^^65^^,^^70^^,^^72^^,^^73^^,^^79^^,^^81^^,^^85^	Not applicable
	Service delivery (n=1)^86^	Costs to the hospital (n=1)^86^
		Procedural characteristics (n=1)^86^
	Other objective tools (n=4)^54^^,^^69^^,^^85^^,^^87^	Symmetry (n=3)^54^^,^^85^^,^^87^
		Function (n=1)^69^

Studies may use multiple outcome measures and therefore may be included more than once.

**Table 5:** Outcome measures used in facial prosthesis research

Theme	Subtheme	Measurement Tool
Patient-reported outcome measures		
Satisfaction	Not applicable	Single-item satisfaction scale^5^^,^^31^, ^32^, ^33^
		Self-designed condition-specific questionnaires proposed by authors (or source not referenced)^5^^,^^32^^,^^34^, ^35^, ^36^, ^37^, ^38^, ^39^, ^40^, ^41^, ^42^, ^43^, ^44^, ^45^, ^46^, ^47^, ^48^, ^49^, ^50^, ^51^
		Condition-specific questionnaires proposed by or modified from others - Hooper et al. 2005, Chang et al. 2005, Markt and Lemon 2001^52^ - Chang et al. 2005^53^^,^^54^ - Korus et al. 2011^55^ - Questionnaires for partial and complete denture treatment^2^ - Questionnaires proposed by Nobel Biocare^56^ - Instrument proposed by Anderson^10^
		Self-designed data collection (case note review)^57^^,^^58^
Quality of life	Condition specific (specific to facial prostheses)	Toronto Outcome Measure for Craniofacial Prosthetics^4^^,^^5^
		Self-designed condition-specific questionnaire and telephone survey^9^
		Condition-specific questionnaires proposed by/modified from - Sloan et al. 2001^6^^,^^7^ - Martin Deadman, Birmingham^8^
	Condition specific (not specific to facial prostheses)	Functional Rhinoplasty Outcome Inventory-17^59^
		Glasgow Benefit Inventory^39^^,^^60^
		University of Washington Quality of Life^61^^,^^62^
		Rhinoplasty Outcome Evaluation^59^
		Rosenberg Self-Esteem Scale^39^
	Generic	Linear Analog Self-Assessment (LASA)^32^ or LASA-12^5^
		Short Form 8 (SF-8 Health Survey),^5^ SF-12 Health Survey,^39^^,^^63^ or SF-36 Health Survey^4^^,^^59^
		World Health Organization Quality of Life Instrument (WHOQOL-BREF)^64^
Psychological health and well-being	Psychological health	Attention to Positive and Negative Information Scale, Short Form^4^
		Hope Scale^4^
		Hospital Anxiety and Depression Scale^4^
		Life Orientation Test-Revised^4^
		Posttraumatic Growth Inventory^4^
		Satisfaction with Life Scale^4^
		Social Avoidance and Distress Scale^4^
		Cornell Medical Index Questionnaire^65^
		Self-designed condition-specific questionnaires created by authors^32^^,^^65^^,^^67^^,^^68^
		Condition-specific questionnaires modified from Sela and Lowental 1980^66^
	Psychosocial	Childhood Experience Questionnaire^67^
Other patient reported	Preference	Preference for attachment system^44^
	Appearance/function	Nasal Appearance and Function Evaluation Questionnaire^61^
	Function	Self-designed condition-specific question^69^
	Functional comfort	Self-designed condition-specific scale^32^
	Duration of wear	Self-designed data collection (case note review)^70^
	Success	Ability to wear prostheses as desired^41^
Clinician-reported outcome measures		
Clinical evaluation	Not applicable	Incoming Clinical Questionnaire and Outgoing Clinical Questionnaire^5^
		Self-designed condition-specific instruments^4^^,^^71^, ^72^, ^73^
Independent observer-reported outcome measures		
Utility	Not applicable	Standard Gamble^74^
		Visual Analog Scale^74^
		Time Trade Off^74^
Appearance	Not applicable	Modified blepharoplasty scale^75^
Outcome measures assessing multiple perspectives		
Appearance	Not applicable	Self-designed scales for clinician and independent observer^66^ or patient and clinician^76^^,^^77^
Success	Not applicable	Self-designed criteria for success including patient reported and clinical factors^78^
Clinical indicators		
Prosthesis survival	Prosthesis survival/lifespan	Time to replacement^33^^,^^39^^,^^57^^,^^73^^,^^79^, ^80^, ^81^, ^82^, ^83^ Reasons for replacement^33^^,^^39^^,^^73^^,^^79^^,^^80^
	Prosthesis failure	Number of failures (prostheses that are not retained by implants)^84^
Aftercare	Not applicable	Self-designed data collection^8^^,^^33^^,^^44^^,^^79^^,^^80^
Complications	Not applicable	Biological complications^47^^,^^58^^,^^70^^,^^72^^,^^79^^,^^85^
		Technical complications^65^^,^^72^^,^^73^^,^^81^^,^^85^
Service delivery	Costs to the hospital	Cost of the prosthesis, operating room, inpatient hospital stay and miscellaneous costs.^86^
	Procedural characteristics	Number of surgical procedures, length of stay within hospital.^86^
Other objective tools	Symmetry	Direct measurements of distances between insertion points of normal and artificial ears and facial mid-plane.^85^
		Asymmetry index—mean distance between the original and mirrored cloud divided by the diagonal of the bounding box of the face.^87^
		Linear distances between fixed anthropometric landmarks (eye fissure length and height) from a standardized photograph with Adobe Photoshop software.^54^
	Function	Acoustic change—real ear testing with a Real-Ear analyzer^69^

Clinical indicators was the second most common category identified in 22 studies (34%) (Table 4). Themes included prosthesis survival, complications (such as skin reactions), prosthetic aftercare (such as repairs), and service delivery (such as costs). Clinician-reported outcome measures were used in 5 studies (8%) and comprised self-designed instruments to capture outcomes from a clinical perspective. Outcome measures reporting multiple perspectives were used in 4 studies (6%) and captured themes relating to appearance or treatment success. Independent observer-reported outcome measures were used in 2 studies (3%) to evaluate health state utility and appearance.

**Figure 2:**
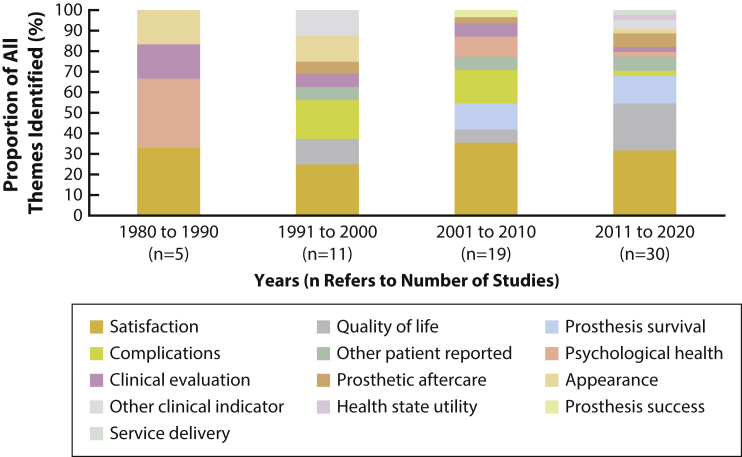
Trends in outcome measure categories used in studies over time.

Figure 2 shows a greater number of outcome measure themes have been used in facial prosthesis research over the decades. Some themes have been used consistently; for example, satisfaction has formed a large proportion of the total themes identified. Other themes such as prosthesis survival have become more popular. Certain themes, for example, health state utility and service delivery, appear to have only recently been evaluated.

## Discussion

Over the past 40 years, facial prosthesis research has focused on PROMs. Clinical indicators was the second most popular category, which is in keeping with the lifelong maintenance and replacement of facial prostheses. New themes have emerged in the literature (such as health state utility and service delivery), which may become increasingly important in the future with focus on delivering clinical and cost-effective services. The increasing thematic variety identified in this systematic review may be due to an increase in the number of studies over time and the clinical and methodologic diversity of the studies.

One key difference between this systematic review and similar published reviews involved the use of quality assessment.^18^^,^^19^ While quality assessment might not be necessary as the review did not synthesize efficacy data,^18^ it was deemed important to provide a holistic overview of the quality of studies in facial prosthesis research. Two of the QATSDD criteria are related to outcome measures: rationale for the choice of data collection tool and statistical assessment of the reliability and validity of the measurement tools.^16^ Both of these criteria did not rate highly among the study designs. This suggests a need for better consideration or reporting of these concepts in the facial prosthesis literature.

A limitation of this systematic review arose from the exclusion of potentially relevant manuscripts where a full-text English-language version was unavailable. This could limit the generalizability of the results if there are variabilities in the choice of outcome measure as a result of language differences. The 40-year inclusion period may have influenced the quality of included studies, as earlier studies may not be subject to recent rigorous reporting criteria. The inclusion period also resulted in challenges when acquiring missing information from earlier publications.

The outcome measure classification system was based on previous reviews.^18^^,^^19^ Selecting the most appropriate categories was a challenge, as some outcome measures were not explicitly defined or related to more than 1 theme. For example, some questionnaires evaluated satisfaction, QoL, self-confidence, and social aspects in a single tool. In addition, concepts such as complications and prosthetic aftercare could overlap. In such situations, categorization was based on the predominant theme and resolved by consensus. The focus of the results may change by undertaking an alternative approach.

Consensus is lacking regarding the most appropriate and meaningful outcome measures to use in facial prosthesis research. For example, patient satisfaction was evaluated by 31 studies; of which, 20 studies created self-designed condition-specific questionnaires, 7 used other authors’ questionnaires (in the original or a modified form), 3 used single-item scales, and 2 collected data from case note review. Condition-specific questionnaires developed and validated for other conditions were found to be used with patients with facial prostheses, and this may not provide meaningful data.

Guidance is available which highlights the ideal features of outcome measures.^88^, ^89^, ^90^ Reliability is the ability to distinguish among individuals despite measurement error,^88^ validity relates to whether the tool measures what it is intended to measure,^90^ and responsiveness refers to whether the tool can distinguish among patients who remain the same, improve, or deteriorate over the course of the study.^88^ The authors recommend that future research uses outcome measures with appropriate measurement properties for use with facial prostheses. Evaluation of measurement properties is beyond the scope of this systematic review^91^; however, condition-specific outcome measures such as the Toronto Outcome Measure for Craniofacial Prosthetics were identified that appeared to be validated in this context.^92^ A standardized set of outcomes may be beneficial to indicate what should be measured and reported in facial prosthesis research.

## Conclusions

Based on the findings of this systematic review, the following conclusions were drawn:

1. The outcome measures reported in the facial prosthesis literature between 1980 and early 2020 have focused on PROMs.
2. An increase in the number of outcome measure themes was identified over time, and concepts such as service delivery and health state utility have recently been evaluated.
3. Future research should use outcome measures with appropriate measurement properties for use with facial prostheses.
4. A standardized set of outcomes for facial prosthetic rehabilitation may guide the development of validated outcome measures to capture the perspectives of different stakeholders.

## References

[1] Ariani N., Visser A., van Oort R.P., Kusdhany L., Rahardjo T.B., Krom B.P. (2013). Current state of craniofacial prosthetic rehabilitation. Int J Prosthodont.

[2] Chang T., Garrett N., Roumanas E., Beumer J. (2005). Treatment satisfaction with facial prostheses. J Prosthet Dent.

[3] Leonardi A., Buonaccorsi S., Pellacchia V., Moricca L.M., Indrizzi E., Fini G. (2008). Maxillofacial prosthetic rehabilitation using extraoral implants. J Craniofac Surg.

[4] Tam C.K., McGrath C.P., Ho S.M.Y., Pow E.H.N., Luk H.W.K., Cheung L.K. (2014). Psychosocial and quality of life outcomes of prosthetic auricular rehabilitation with CAD/CAM technology. Int J Dent.

[5] Kiat-amnuay S., Jacob R.F., Chambers M.S., Anderson J.D., Sheppard R.A., Johnston D.A. (2010). Clinical trial of chlorinated polyethylene for facial prosthetics. Int J Prosthodont.

[6] Nemli S.K., Aydin C., Yilmaz H., Bal B.T., Arici Y.K. (2013). Quality of life of patients with implant-retained maxillofacial prostheses: a prospective and retrospective study. J Prosthet Dent.

[7] de Oliveira F.M., Salazar-Gamarra R., Ohman D., Nannmark U., Pecorari V., Dib L.L. (2018). Quality of life assessment of patients utilizing orbital implant-supported prostheses. Clin Implant Dent Relat Res.

[8] Westin T., Tjellström A., Hammerlid E., Bergström K., Rangert B. (1999). Long-term study of quality and safety of osseointegration for the retention of auricular prostheses. Otolaryngol Head Neck Surg.

[9] Tolman D.E., Taylor P.F. (1996). Bone-anchored craniofacial prosthesis study. Int J Oral Maxillofac Implants.

[10] Rotenberg B.W., James A.L., Fisher D., Anderson J., Papsin B.C. (2002). Establishment of a bone-anchored auricular prosthesis (BAAP) program. Int J Pediatr Otorhinolaryngol.

[11] Liacouras P., Garnes J., Roman N., Petrich A., Grant G.T. (2011). Designing and manufacturing an auricular prosthesis using computed tomography, 3-dimensional photographic imaging, and additive manufacturing: a clinical report. J Prosthet Dent.

[12] Hatamleh M.M., Haylock C., Watson J., Watts D.C. (2010). Maxillofacial prosthetic rehabilitation in the UK: a survey of maxillofacial prosthetists' and technologists' attitudes and opinions. Int J Oral Maxillofac Surg.

[13] Sloan J.A., Tolman D.E., Anderson J.D., Sugar A.W., Wolfaardt J.F., Novotny P. (2001). Patients with reconstruction of craniofacial or intraoral defects: development of instruments to measure quality of life. Int J Oral Maxillofac Implants.

[14] Penkner K., Santler G., Mayer W., Pierer G., Lorenzoni M. (1999). Fabricating auricular prostheses using three-dimensional soft tissue models. J Prosthet Dent.

[15] Farook T.H., Jamayet N.B., Abdullah J.Y., Rajion Z.A., Alam M.K. (2020). A systematic review of the computerized tools and digital techniques applied to fabricate nasal, auricular, orbital and ocular prostheses for facial defect rehabilitation. J Stomatol Oral Maxillofac Surg.

[16] Sirriyeh R., Lawton R., Gardner P., Armitage G. (2012). Reviewing studies with diverse designs: the development and evaluation of a new tool. J Eval Clin Pract.

[17] Higgins J.P.T., Thomas J., Chandler J., Cumpston M., Li T., Page M.J. (2019). Cochrane handbook for systematic reviews of interventions version 6.0 (updated July 2019).

[18] Barber S., Bekker H.L., Meads D., Pavitt S., Khambay B. (2018). Identification and appraisal of outcome measures used to evaluate hypodontia care: a systematic review. Am J Orthod Dentofacial Orthop.

[19] Levey C., Innes N., Schwendicke F., Lamont T., Gostemeyer G. (2017). Outcomes in randomised controlled trials in prevention and management of carious lesions: a systematic review. Trials.

[20] Jablonski R., Veale B., Coward T., Keeling A., Bojke C., Pavitt S. (2019).

[21] Lefebvre C., Glanville J., Briscoe S., Littlewood A., Marshall C., Metzendorf M.-I., Higgins J.P.T., Thomas J., Chandler J., Cumpston M., Li T., Page M.J. (2019). Cochrane handbook for systematic reviews of interventions version 6.0 (updated July 2019).

[22] Li T., Higgins J.P.T., Deeks J.J., Higgins J.P.T., Thomas J., Chandler J., Cumpston M., Li T., Page M.J. (2019). Cochrane handbook for systematic reviews of interventions version 60 (updated July 2019).

[23] Wallace A., Rogers H.J., Zaitoun H., Rodd H.D., Gilchrist F., Marshman Z. (2017). Traumatic dental injury research: on children or with children?. Dent Traumatol.

[24] Knapp R., Gilchrist F., Rodd H.D., Marshman Z. (2017). Change in children's oral health-related quality of life following dental treatment under general anaesthesia for the management of dental caries: a systematic review. Int J Paediatr Dent.

[25] Aliakbari E., Gray-Burrows K.A., Vinall-Collier K.A., Edwebi S., Marshman Z., McEachan R.R.C. (2021). Home-based toothbrushing interventions for parents of young children to reduce dental caries: A systematic review. Int J Paediatr Dent.

[26] Fenton L., Lauckner H., Gilbert R. (2015). The QATSDD critical appraisal tool: comments and critiques. J Eval Clin Pract.

[27] Anderson J.D., Johnston D.A., Haugh G.S., Kiat-Amnuay S., Gettleman L. (2013). The Toronto outcome measure for craniofacial prosthetics: reliability and validity of a condition-specific quality-of-life instrument. Int J Oral Maxillofac Implants.

[28] Becker C., Becker A.M., Pfeiffer J. (2016). Health-related quality of life in patients with nasal prosthesis. J Craniomaxillofac Surg.

[29] Mevio E., Facca L., Mullace M., Sbrocca M., Gorini E., Artesi L. (2015). Osseointegrated implants in patients with auricular defects: a case series study. Acta Otorhinolaryngol Ital.

[30] Tolman D.E. (1998). Reconstructing the human face. J Facial Somato Prosthet.

[31] Nassab R.S., Thomas S.S., Murray D. (2007). Orbital exenteration for advanced periorbital skin cancers: 20 years experience. J Plast Reconstr Aesthet Surg.

[32] Schoen P.J., Raghoebar G.M., van Oort R.P., Reintsema H., van der Laan B., Burlage F.R. (2001). Treatment outcome of bone-anchored craniofacial prostheses after tumor surgery. Cancer.

[33] Korfage A., Raghoebar G.M., Noorda W.D., Plaat B.E., Vissink A., Visser A. (2016). Recommendations for implant-retained nasal prostheses after ablative tumor surgery: minimal surgical aftercare, high implant survival, and satisfied patients. Head Neck.

[34] Jebreil K. (1980). Acceptability of orbital prostheses. J Prosthet Dent.

[35] Chen M.S., Udagama A., Drane J.B. (1981). Evaluation of facial prostheses for head and neck cancer patients. J Prosthet Dent.

[36] Xu X., Wang C., Liu S., Deng Y. (1997). Designing manufacturing and the clinic application of the prosthesis for the patients with large ocular defects. Yan Ke Xue Bao.

[37] Younis I., Gault D., Sabbagh W., Kang N.V. (2010). Patient satisfaction and aesthetic outcomes after ear reconstruction with a Branemark-type, bone-anchored, ear prosthesis: a 16 year review. J Plast Reconstr Aesthet Surg.

[38] Korus L.J., Wong J.N., Wilkes G.H. (2011). Long-term follow-up of osseointegrated auricular reconstruction. Plast Reconstr Surg.

[39] Kievit H., Verhage-Damen G.W.J.A., Ingels K.J., Mylanus E.A.M., Hol M.K.S. (2013). Long-term quality of life assessment in patients with auricular prostheses. J Craniofac Surg.

[40] Wondergem M., Lieben G., Bouman S., van den Brekel M.W.M., Lohuis P.J.F.M. (2016). Patients' satisfaction with facial prostheses. Br J Oral Maxillofac Surg.

[41] Zuo K.J., Wilkes G.H. (2016). Clinical outcomes of osseointegrated prosthetic auricular reconstruction in patients with a compromised ipsilateral temporoparietal fascial flap. J Craniofac Surg.

[42] Dings J.P.J., Merkx M.A.W., de Clonie Maclennan-Naphausen M.T.P., van de Pol P., Maal TJJ, Meijer G.J. (2018). Maxillofacial prosthetic rehabilitation: a survey on the quality of life. J Prosthet Dent.

[43] Agarwal C.A., Johns D., Tanner P.B., Andtbacka R.H.I. (2018). Osseointegrated prosthetic ear reconstruction in cases of skin malignancy: technique, outcomes, and patient satisfaction. Ann Plast Surg.

[44] Visser A., Noorda W.D., Linde A., Raghoebar G.M., Vissink A. (2020). Bar-clip versus magnet-retained auricular prostheses: a prospective clinical study with a 3-year follow-up. J Prosthet Dent.

[45] Kang N.V., Morritt D., Pendegrass C., Blunn G. (2013). Use of ITAP implants for prosthetic reconstruction of extra-oral craniofacial defects. J Plast Reconstr Aesthet Surg.

[46] Stevenson D.S., Proops D.W., Wake M.J.C., Deadman M.J., Worrollo S.J., Hobson J.A. (1993). Osseointegrated implants in the management of childhood ear abnormalities: the initial Birmingham experience. J Laryngol Otol.

[47] Jacobsson M., Tjellstrom A., Fine L., Jansson K. (1992). An evaluation of auricular prostheses using osseointegrated implants. Clin Otolaryngol Allied Sci.

[48] Markt J.C., Lemon J.C. (2001). Extraoral maxillofacial prosthetic rehabilitation at the M. D. Anderson Cancer Center: a survey of patient attitudes and opinions. J Prosthet Dent.

[49] Wagenblast J., Baghi M., Helbig M., Arnoldner C., Bisdas S., Gstöttner W. (2008). Craniofacial reconstructions with bone-anchored epithesis in head and neck cancer patients--a valid way back to self-perception and social reintegration. Anticancer Res.

[50] Hooper S.M., Westcott T., Evans P.L., Bocca A.P., Jagger D.C. (2005). Implant-supported facial prostheses provided by a maxillofacial unit in a U.K. regional hospital: longevity and patient opinions. J Prosthodont.

[51] Smolarz-Wojnowska A., Raithel F., Gellrich N.C., Klein C. (2014). Quality of implant anchored craniofacial and intraoral prostheses: patient's evaluation. J Craniofac Surg.

[52] Karakoca Nemli S., Aydin C., Yilmaz H., Sarisoy S., Kasko Y. (2010). Retrospective study of implant-retained orbital prostheses: implant survival and patient satisfaction. J Craniofac Surg.

[53] Sandner A., Bloching M. (2009). Retrospective analysis of titanium plate-retained prostheses placed after total rhinectomy. Int J Oral Maxillofac Implants.

[54] Abd El Salam S.E., Eskandar A.E., Mohammed K.A. (2020). Patient satisfaction of orbital prosthesis fabricated by the aid of rapid prototyping technology versus conventional technique in orbital defect patients: a crossover randomized clinical trial. Int J Maxillofac Prosthetics.

[55] DeSerres J.J., Budden C.R., Wolfaardt J.F., Wilkes G.H. (2017). Long-term follow-up of osseointegrated orbital prosthetic reconstruction. J Craniofac Surg.

[56] Arcuri M.R., LaVelle W.E., Fyler A., Funk G. (1997). Effects of implant anchorage on midface prostheses. J Prosthet Dent.

[57] Si Y., Fan S.C., Sun W., Chen Y.B., Zhang Z.G. (2012). Osseointegration technique in patients with acquired auricular deformities and failed previous reconstruction: a retrospective study of long-term follow-up and Chinese experience. ORL J Otorhinolaryngol Relat Spec.

[58] Papaspyrou G., Yildiz C., Bozzato V., Bohr C., Schneider M., Hecker D. (2018). Prosthetic supply of facial defects: long-term experience and retrospective analysis on 99 patients. Eur Arch Otorhinolaryngol.

[59] Zaoui K., Thielen H.M., Plath M., Baumann I., Plinkert P.K., Federspil P.A. (2018). Quality of life after nasal cancer resection - surgical versus prosthetic rehabilitation. Rhinology.

[60] Vijverberg M.A., Verhamme L., van de Pol P., Kunst H.P.M., Mylanus E.A.M., Hol M.K.S. (2019). Auricular prostheses attached to osseointegrated implants: multidisciplinary work-up and clinical evaluation. Eur Arch Otorhinolaryngol.

[61] Becker C., Becker A.M., Dahlem K.K.K., Offergeld C., Pfeiffer J. (2017). Aesthetic and functional outcomes in patients with a nasal prosthesis. Int J Oral Maxillofac Surg.

[62] Worrell E., Worrell L., Bisase B. (2017). Care of long-term survivors of head and neck cancer after treatment with oral or facial prostheses, or both. Br J Oral Maxillofac Surg.

[63] Mevio E., Facca L., Schettini S., Mullace M. (2016). Bone-anchored titanium implants in patients with auricular defects: three years and 27 patients' experience. Int J Otolaryngol.

[64] Atay A., Peker K., Gunay Y., Ebrinc S., Karayazgan B., Uysal O. (2013). Assessment of health-related quality of life in Turkish patients with facial prostheses. Health Qual Life Outcomes.

[65] Honda M.J., Hatanaka T., Okazaki Y., Ueda M. (2005). Long-term results of osseointegrated implant-retained facial prostheses: a 5-year retrospective study. Nagoya J Med Sci.

[66] Lowental U., Sela M. (1982). Evaluating cosmetic results in maxillofacial prosthetics. J Prosthet Dent.

[67] Horlock N., Vögelin E., Bradbury E.T., Grobbelaar A.O., Gault D.T. (2005). Psychosocial outcome of patients after ear reconstruction: a retrospective study of 62 patients. Ann Plast Surg.

[68] Sela M., Lowental U. (1980). Therapeutic effects of maxillofacial prostheses. Oral Surg Oral Med Oral Pathol.

[69] Reisberg D.J., Lipner M. (1993). Audiometric evaluation of prosthetic ears: a preliminary report. J Prosthet Dent.

[70] Hamming K.K., Lund T.W., Lander T.A., Sidman J.D. (2009). Complications and satisfaction with pediatric osseointegrated external ear prostheses. Laryngoscope.

[71] Roefs A.J., van Oort R.P., Schaub R.M. (1984). Factors related to the acceptance of facial prostheses. J Prosthet Dent.

[72] Berg A., Stark B., Larson O., Blomgren I., Edstrom K., Wilson R. (1994). Four-year experience with titanium implants for cranio-facial rehabilitation in plastic surgery. Eur J Plast Surg.

[73] Karakoca S., Aydin C., Yilmaz H., Bal B.T. (2010). Retrospective study of treatment outcomes with implant-retained extraoral prostheses: survival rates and prosthetic complications. J Prosthet Dent.

[74] Faris C., Heiser A., Quatela O., Jackson M., Tessler O., Jowett N. (2020). Health utility of rhinectomy, surgical nasal reconstruction, and prosthetic rehabilitation. Laryngoscope.

[75] Kuiper J.J., Zimmerman M.B., Pagedar N.A., Carter K.D., Allen R.C., Shriver E.M. (2016). Perception of patient appearance following various methods of reconstruction after orbital exenteration. Orbit.

[76] Granstrom G., Bergstrom K., Tjellstrom A. (1993). The bone-anchored hearing-aid and bone-anchored epithesis for congenital ear malformations. Otolaryngol Head Neck Surg.

[77] Keerl R., Weber R., Scholtes W., Draf W., Heieis G., Trainer D. (1996). Prosthetic rehabilitation after craniofacial surgery. Skull Base Surg.

[78] Wright R.F., Zemnick C., Wazen J.J., Asher E. (2008). Osseointegrated implants and auricular defects: a case series study. J Prosthodont.

[79] Visser A., Raghoebar G.M., Van Oort R.P., Vissink A. (2008). Fate of implant-retained craniofacial prostheses: life span and aftercare. Int J Oral Maxillofac Implants.

[80] Brandão T.B., Vechiato Filho A.J., de Souza Batista V.E., Prado Ribeiro A.C., Filho H.N., Chilvarquer I. (2017). Assessment of treatment outcomes for facial prostheses in patients with craniofacial defects: a pilot retrospective study. J Prosthet Dent.

[81] Aydin C., Karakoca S., Yilmaz H., Yilmaz C. (2008). Implant-retained auricular prostheses: an assessment of implant success and prosthetic complications. Int J Prosthodont.

[82] Subramaniam S.S., Breik O., Cadd B., Peart G., Wiesenfeld D., Heggie A. (2018). Long-term outcomes of craniofacial implants for the restoration of facial defects. Int J Oral Maxillofac Surg.

[83] Curi M.M., Oliveira M.F., Molina G., Cardoso C.L., De Groot Oliveira L., Branemark P.I. (2012). Extraoral implants in the rehabilitation of craniofacial defects: implant and prosthesis survival rates and peri-implant soft tissue evaluation. J Oral Maxillofac Surg.

[84] Ethunandan M., Downie I., Flood T. (2010). Implant-retained nasal prosthesis for reconstruction of large rhinectomy defects: the Salisbury experience. Int J Oral Maxillofac Surg.

[85] Watson R.M., Coward T.J., Forman G.H. (1995). Results of treatment of 20 patients with implant-retained auricular prostheses. Int J Oral Maxillofac Implants.

[86] Ryan M.A., Khoury T., Kaylie D.M., Crowson M.G., Brown C.S., McClennen J. (2018). Osseointegrated implants for auricular prostheses: an alternative to autologous repair. Laryngoscope.

[87] Bockey S., Berssenbrugge P., Dirksen D., Wermker K., Klein M., Runte C. (2018). Computer-aided design of facial prostheses by means of 3D-data acquisition and following symmetry analysis. J Craniomaxillofac Surg.

[88] Johnston B.C., Patrick D.L., Devji T., Maxwell L.J., Bingham C.O., Beaton D., Higgins J.P.T., Thomas J., Chandler J., Cumpston M., Li T., Page M.J. (2019). Cochrane handbook for systematic reviews of interventions version 6.0 (updated July 2019).

[89] Anderson J.D. (1998). The need for criteria on reporting treatment outcomes. J Prosthet Dent.

[90] Jerosch-Herold C. (2005). An evidence-based approach to choosing outcome measures: a checklist for the critical appraisal of validity, reliability and responsiveness studies. Brit J Occup Ther.

[91] Prinsen C.A.C., Mokkink L.B., Bouter L.M., Alonso J., Patrick D.L., de Vet H.C.W. (2018). COSMIN guideline for systematic reviews of patient-reported outcome measures. Qual Life Res.

[92] Anderson J.D., Szalai J.P. (2003). The Toronto outcome measure for craniofacial prosthetics: a condition-specific quality-of-life instrument. Int J Oral Maxillofac Implants.

