# Supplementary material for: Outcome measures in facial prosthesis research: A systematic review
Source: J Prosthet Dent. 2021 Dec;126(6):805–15. doi: 10.1016/j.prosdent.2020.09.010 (PMC8664412; doi:10.1016/j.prosdent.2020.09.010)
Supplement: Supplementary Tables [file mmc1.docx]

**SUPPLEMENTARY TABLES**

Supplemental Table 1. Study characteristics for experimental studies

| **Study** | **Setting** | **Design** | **Ages**  **Defect**  **Etiology** | **Intervention** | **Comparator** | **Participant numbers** | **Category** | **Measurement tool** | **Reference for tool** | **Tool modified** | **Validation reported** |
| --- | --- | --- | --- | --- | --- | --- | --- | --- | --- | --- | --- |
| Abd El Salam et al, 2020^54^ | Egypt | Cross-over RCT | Ad, Ch  Orb  Onc | FP CAD-CAM manufacture | FP conventional manufacture | 8 | Satisfaction | Condition-specific questionnaire proposed by Chang et al, 2005 | Yes | Yes without explanation | No |
|  |  |  |  |  |  |  | Objective tool - dimensional accuracy | Linear distances (eye fissure length and height) from a standardized photograph. | Yes | N/A | Not explicit |
| Kiat-amnuay et al, 2010^5^ / Anderson et al, 2013^27^ | USA and Canada | Cross-over RCT | Ad  Aur, Nas, Orb  Con, Onc, Tra | Adhesive retained FP, chlorinated polyethylene elastomer | Adhesive retained FP, silicone | 42 (28 completed study) | Clinician evaluation | Incoming Clinical Questionnaire and Outgoing Clinical Questionnaire | No | N/A | No |
|  |  |  |  |  |  |  | Quality of life (condition specific) | Toronto Outcome Measure for Craniofacial Prosthetics (52 item version for previous prosthesis users and 29 item for new users) | Yes | No | Yes |
|  |  |  |  |  |  |  | Quality of Life (generic) | Linear Analogue Self-Assessment LASA-12 | No - refers to source | No | Not explicit |
|  |  |  |  |  |  |  | Quality of life (generic) | Short Form 8 (SF-8 Health Survey) | Yes | No | Not explicit |
|  |  |  |  |  |  |  | Satisfaction | Patient Evaluation Questionnaire | No | N/A | No |
|  |  |  |  |  |  |  |  | Exit Questionnaire | No | N/A | No |

Ad, adult > 18 years of age; Ch, child < 18 years of age; Aur, auricular; Nas, nasal; Orb, orbital; Ocu, ocular; Com, combined; IO, intraoral; Con, congenital; Onc, oncology; Tra, trauma; Inf, infection; Bur, burns; Exe, exenteration (reason not specified); FP, facial prosthesis; CAD-CAM, computer-aided design and computer-aided manufacturing; recon, reconstruction; RR, response rate; N/S, not specified; N/A, not applicable

Supplemental Table 2. Study characteristics for cross-sectional studies

| **Study** | **Setting** | **Design** | **Ages**  **Defect**  **Etiology** | **Intervention** | **Comparator** | **Participant numbers** | **Category** | **Measurement tool** | **Reference for tool** | **Tool modified** | **Validation reported** |
| --- | --- | --- | --- | --- | --- | --- | --- | --- | --- | --- | --- |
| de Oliveira et al, 2018^7^ | Brazil | Cross-sectional, observational, descriptive | Ad  Orb  Onc, Tra, Inf | Implant retained FP | N/A | 45.  RR not explicit | Quality of life | Condition-specific questionnaire proposed by Sloan et al, 2001 | Yes | Yes - with explanation | Yes pre-modifications |
| Dings et al, 2018^42^ | The Netherlands | Cross-sectional, observational, analytical | Ad  Nas, Aur, Orb  Con, Onc, Tra, N/S | Implant retained FP | Adhesive retained FP | 66.  RR = 52/66 (79%). | Satisfaction | Self-designed condition-specific questionnaire | No | N/A | No |
| Becker et al, 2017^61^ / Becker et al, 2016^28^ | Germany | Cross-sectional, observational, descriptive | Ad  Nas  Onc | Implant retained FP | N/A | 43.  RR not explicit. | Esthetic and functional outcomes | Nasal Appearance and Function Evaluation Questionnaire (NAFEQ) (German version) (used in surgical nasal reconstruction) | Yes | Yes - with explanation | In other contexts |
|  |  |  |  |  |  |  | Quality of Life (condition specific non-FP) | University of Washington Quality of Life UW-QoL (German version) (used in head and neck cancer) | Yes | No | No |
| Kuiper et al, 2016^75^ | USA | Cross-sectional, observational, analytical | N/S  Orb  Exe | FP | 3 surgical recon techniques | 607 independent observers. RR= 125/607 (21%) completed all questions. | Appearance | Blepharoplasty Scale | Yes | Yes - without explanation | No |
| Wondergem et al, 2016^40^ | The Netherlands | Cross-sectional, observational, analytical | Ad  Nas, Aur, Orb, Com  Con, Onc, Tra, benign | Implant retained FP | Adhesive retained FP | 104.  RR = 71/104 (68%) | Satisfaction | Self-designed condition-specific questionnaire | No | N/A | No |
| Smolarz-Wojnowska et al, 2014^51^ | Germany | Cross-sectional, observational, analytical | Ad  Nas, Aur, Orb, IO, partial face  Onc | Implant retained FP | Previous adhesive/ mechanically retained FP | 30 (26 with FP).  RR not explicit. | Satisfaction | Self-designed condition-specific questionnaire | No | N/A | No |
| Atay et al, 2013^64^ | Turkey | Cross-sectional, observational, analytical | Ad  Nas, Aur, Orb  Onc, Tra | Implant and adhesive retained FP | Healthy controls without any deformity | 72 FP plus 24 healthy controls.  No missing data reported. | Quality of life (generic) | World Health Organisation Quality of Life Instrument (WHOQOL-BREF) (Turkish version) | Yes | No | In other contexts |
| Chang et al, 2005^2^ | USA | Cross-sectional, observational, analytical | Ad  Nas, Aur, Orb  Con, Onc, Tra | Implant retained FP | Adhesive retained FP | 35.  RR not explicit. | Satisfaction | Condition-specific questionnaire based on questionnaires for partial and complete denture treatment | Yes | Yes with explanation | No |
| Hooper et al, 2005^50^ | UK | Cross-sectional, observational, descriptive | Ad, Ch  Nas, Aur, Orb, Com  N/S | FP | N/A | 75.  RR = 47/75 (63%) | Satisfaction | Self-designed condition-specific questionnaire | No | N/A | No |
| Horlock et al, 2005^67^ | UK | Cross-sectional, observational, analytical | Ad, Ch  Aur  Con, Onc, Tra, Inf | Autologous reconstruction or FP | Unaffected school children evaluated in previous study | 108 autologous and 12 FP. RR = 62/90 (69%). | Psychological health | Self-designed condition-specific questionnaire | No | N/A | No |
|  |  |  |  |  |  |  | Psychosocial | Childhood Experience Questionnaire (under 12s only) (used in craniofacial deformity and surgical reconstruction) | Yes | No | No |
| Markt and Lemon 2001^48^ | USA | Cross-sectional, observational, descriptive | Ad  Nas, Aur, Orb, Ocu, Com, IO, N/S, toe  Onc, non-neoplastic | Adhesive and mechanically retained FP | N/A | 263.  RR = 76/263 (29%) | Satisfaction | Self-designed condition-specific questionnaire | No | N/A | No |
| Jacobsson et al, 1992^47^ | Sweden | Cross-sectional, observational, analytical | Ad, Ch  Aur  Con, Onc, Tra | Implant retained FP, conventional manufacture | Previous FP, conventional retention | 49.  RR= 47/49 (96%). | Satisfaction | Self-designed condition-specific questionnaire | No | N/A | No |
|  |  |  |  |  |  |  | Complications | Numbers of biological complications (confirmed by means of case note review) |  |  |  |
| Chen et al, 1981^35^ | USA | Cross-sectional, observational, descriptive | Ad, Ch  Nas, Aur, Orb, Com, cheek  N/S (likely all onc) | FP | N/A | 138.  RR = 73/138 (53%) | Satisfaction | Self-designed condition-specific questionnaire | No | N/A | No |
| Jebreil 1980^34^ | USA | Cross-sectional, observational, descriptive | Ad, N/S  Orb  Onc, Tra | Adhesive or mechanically retained FP or eye patch | N/A | 27.  RR = 18/27 (67%) | Satisfaction | Self-designed condition-specific questionnaire | No | N/A | No |

Supplemental Table 3. Study characteristics for prospective longitudinal observational studies

| **Study** | **Setting** | **Design** | **Ages**  **Defect**  **Etiology** | **Intervention** | **Comparator** | **Participant numbers** | **Category** | **Measurement tool** | **Reference for tool** | **Tool modified** | **Validation reported** |
| --- | --- | --- | --- | --- | --- | --- | --- | --- | --- | --- | --- |
| Faris et al, 2020^74^ | USA | Prospective, observational, analytical | Ad  Nas  N/S (likely all onc) | Clinical vignette of an implant retained FP | Clinical vignette of a surgical reconstruction | 310 naive observers. 37 surveys excluded. RR = 88% | Health state utility assessment | Visual Analogue Scale | Yes | No | Not explicit |
|  |  |  |  |  |  |  |  | Standard Gamble | Yes | No | Not explicit |
|  |  |  |  |  |  |  |  | Time Trade Off | Yes | No | Not explicit |
| Visser et al, 2020^44^ | The Netherlands | Prospective, observational, analytical | Ad  Aur  Con, Onc, Tra | Implant (magnet) retained FP conventional manufacture | Compared to previous bar-clip retained FP | 17.  RR = 1 lost to follow up at 3 years. | Preference | Preference for attachment system | No | N/A | No |
|  |  |  |  |  |  |  | Satisfaction | Self-designed condition-specific questionnaire | No | N/A | No |
|  |  |  |  |  |  |  | Prosthetic aftercare | Number of aftercare procedures | No | N/A | No |
| Zaoui et al, 2018^59^ | Germany | Prospective, observational, analytical | Ad  Nas  Onc | Implant retained FP | Surgical recon | 64 (27 FP, 37 surgical recon).  51 alive at data collection. RR =32/51 (63%). | Quality of life (condition specific non FP) | Functional Rhinoplasty Outcome Inventory-17 (FROI-17) | Yes | No | In other contexts |
|  |  |  |  |  |  |  |  | Rhinoplasty Outcome Evaluation (ROE) | Yes | No | In other contexts |
|  |  |  |  |  |  |  | Quality of life (generic) | Short Form 36 (SF-36 Health Survey) | Yes | No | Not explicit |
| Mevio et al, 2016^63^ / Mevio et al, 2015^29^ | Italy | Prospective, observational, descriptive | Ad, Ch  Aur  Con, Onc, Tra, Bur | Implant retained FP, conventional manufacture | N/A | 27.  RR not explicit | Quality of life (generic) | Short Form 12 (SF-12 Health Survey) | Yes | No | No |
| Tam et al, 2014^4^ | China | Prospective, observational, descriptive | Ad  Aur  Con | Implant retained FP, CAD-CAM manufacture | N/A | 6.  RR = 50% included at 1 year | Quality of life (generic) | Short Form 36 (SF-36 Health Survey) | Yes | No | No |
|  |  |  |  |  |  |  | Quality of life (condition specific) | Toronto Outcome Measure for Craniofacial Prosthetics (Chinese version) (52 item version) | Yes | No | Yes |
|  |  |  |  |  |  |  | Psychological health | Attention to Positive and Negative Information (APNI) Scale, Short Form | Yes | No | No |
|  |  |  |  |  |  |  |  | Hope Scale (HS) | Yes | No | No |
|  |  |  |  |  |  |  |  | Hospital Anxiety and Depression Scale (HADS) | Yes | No | No |
|  |  |  |  |  |  |  |  | Life Orientation Test-Revised (LOT-R) | Yes | No | No |
|  |  |  |  |  |  |  |  | Posttraumatic Growth Inventory | Yes | No | No |
|  |  |  |  |  |  |  |  | Satisfaction with Life Scale (SWLS) | Yes | No | No |
|  |  |  |  |  |  |  |  | Social Avoidance and Distress (SAD) Scale | Yes | No | No |
|  |  |  |  |  |  |  | Clinician evaluation | Self-designed condition-specific questionnaire | No | N/A | No |
| Nemli et al, 2013^6^ | Turkey | Prospective, retrospective, observational, analytical | Ad, Ch  Nas, Aur, Orb  Con, Onc, Tra, Bur | Implant retained FP, conventional manufacture (bar-clip vs magnet) | Covering or FP with conventional retention. | 82.  RR not explicit. | Quality of life (condition specific) | Condition-specific questionnaire proposed by Sloan et al, 2001 | Yes | Yes - with explanation | Yes pre-modifications |
| Aydin et al, 2008^81^ | Turkey | Prospective, observational, descriptive | Ad, Ch  Aur  Con, Tra, Bur | Implant retained FP | N/A | 10 | Prosthesis survival | Time to replacement | No | N/A | No |
|  |  |  |  |  |  |  | Complications | Numbers of technical complications |  |  |  |
| Arcuri et al, 1997^56^ | USA | Prospective, observational, analytical | Ad  Nas, Com, IO  Onc, Tra | Implant retained FP | Previous covering or FP with conventional retention | 5 (4 had FP).  RR = 80%/100% pre/post-op | Satisfaction | Condition-specific questionnaire proposed by Nobel Biocare | No | N/A | No |
| Xu et al, 1997^36^ | China | Prospective, observational, descriptive | N/S  Orb, Ocu  Tra, Exe | Mechanically retained FP, conventional manufacture | N/A | 50.  RR rate not explicit | Satisfaction | Self-designed condition-specific questionnaire | No | N/A | No |
| Tolman and Taylor 1996^9^ / Tolman 1998^30^ | USA | Prospective, observational, descriptive | Ad, Ch  Nas, Aur, Orb  N/S | Implant retained FP | N/A | 145.  RR = 115 examined throughout entire study | Quality of Life (condition specific) | Self-designed condition-specific questionnaire and telephone survey | No | N/A | No |
| Watson et al, 1995^85^ | UK | Prospective, observational, descriptive | Ad, Ch  Aur  Con, Tra | Implant retained FP, conventional manufacture | N/A | 20 | Objective measurement tool- asymmetry | Direct measurements of distances between insertion points of normal and artificial ears and facial mid-plane. | No | N/A | No |
|  |  |  |  |  |  |  | Complications | Numbers of biological and technical complications |  |  |  |
| Reisberg and Lipner 1993^69^ | USA | Prospective, observational, analytical | Ad, Ch  Aur  Onc, Tra | With FP in situ | Without FP in situ | 4 | Objective measurement tool - function | Acoustic change - real ear testing with a Real-Ear analyzer) | No | N/A | No |
|  |  |  |  |  |  |  | Function | Self-designed condition-specific question | No | N/A | No |
| Lowental and Sela 1982^66^ | Israel | Prospective, observational, descriptive | Ad, Ch  N/S  Con, Onc, Tra | FP | N/A | 52.  RR not explicit | Psychological health | Condition-specific questionnaire by Sela and Lowenta 1980 | Yes | Yes - with some explanation | No |
|  |  |  |  |  |  |  | Appearance | Self-designed 5 grade scale to assess the initial facial defect and the final result by maxillofacial prosthodontist, physician from another hospital and a professional sculptor. | No | N/A | No |
| Sela and Lowental 1980^68^ | Israel | Prospective, observational, descriptive | Ad, Ch  N/S  Con, Onc, Tra | FP | N/A | 56.  RR = 39/56 (70%) | Psychological health | Self-designed condition-specific questionnaire | No | N/A | No |

Supplemental Table 4. Study characteristics for retrospective longitudinal observational studies

| **Study** | **Setting** | **Design** | **Ages**  **Defect**  **Etiology** | **Intervention** | **Comparator** | **Participant numbers** | **Category** | **Measurement tool** | **Reference for tool** | **Tool modified** | **Validation reported** |
| --- | --- | --- | --- | --- | --- | --- | --- | --- | --- | --- | --- |
| Vijverberg et al, 2019^60^ | The Netherlands | Retrospective, observational, descriptive | Ad, Ch  Aur  Con, Onc, Tra | Implant retained FP, CAD-CAM | N/A | 11.  RR = 10/11 (91%) | Quality of life (condition specific non FP) | Glasgow Benefit Inventory (GBI) (used in Otolaryngological interventions) | Yes | No | No |
| Agarwal et al, 2018^43^ | USA | Retrospective, observational, descriptive | Ad  Aur  Onc | Implant retained FP, CAD-CAM | N/A | 21  RR = 15/21 (71%) | Satisfaction | Self-designed condition-specific questionnaire | No | N/A | No |
| Bockey et al, 2018^87^ | Germany | Retrospective, observational, analytical | Ad  Orb  Onc, Tra | CAD template (prosthesis not made) | Compared to mirrored eye, cut defect and conventional FP | 32 (23 had a FP). | Objective measurement tool - facial symmetry | Asymmetry Index - mean distance between the original and mirrored cloud divided by the diagonal of the bounding box of the face | Yes | N/A | In other contexts |
| Papaspyrou et al, 2018^58^ | Germany | Retrospective, observational, descriptive | N/S  Nas, Aur, Orb  Con, Onc, Tra, Inf | Adhesive, implant or mechanically retained FP | N/A | 99. | Satisfaction | Self-designed data collection (case note review) | No | N/A | No |
|  |  |  |  |  |  |  | Complications | Numbers of biological complications |  |  |  |
| Ryan et al, 2018^86^ | USA | Retrospective observational, analytical | Ad, Ch  Aur  Con, Onc, Tra | Implant retained FP | Autologous repairs | 25 (16 FP, 9 surgical repairs) | Service delivery | Costs to the hospital (cost of the FP, operating room, inpatient hospital stay and miscellaneous costs) | No | N/A | No |
|  |  |  |  |  |  |  |  | Procedural characteristics (Number of surgical procedures, length of stay within hospital) | No | N/A | No |
| Subramaniam et al, 2018^82^ | Australia | Retrospective, observational, descriptive | Ad, Ch  Nas, Aur, Orb  Con, Onc, Tra, Inf | Implant retained FP | N/A | 110 participants (1 had eye shield) | Prosthesis survival | Time to replacement | No | N/A | No |
| Brandão et al, 2017^80^ | Brazil | Retrospective, observational, descriptive | Ad, Ch  Nas, Aur, Orb  Con, Onc, Tra | Adhesive or implant retained FP, conventional manufacture | N/A | 27 | Prosthesis survival | Time to replacement and reason for replacement | No | N/A | No |
|  |  |  |  |  |  |  | Prosthetic aftercare | Number of aftercare procedures | No | N/A | No |
| DeSerres et al, 2017^55^ | Canada | Retrospective, observational, descriptive | Ad, Ch  Orb  Con, Onc, Tra | Implant retained FP, CAD-CAM | N/A | 26. 19 sent survey.  RR= 11/19 (58%) | Satisfaction | Condition-specific questionnaire proposed by Korus et al. 2011 | Yes | No | No |
| Korfage et al, 2016^33^ | The Netherlands | Retrospective  observational, descriptive | N/S  Nas  Onc | Implant retained FP | N/A | 28 | Satisfaction | 10-point scale by Schoen et al. 2001 | Yes | No | No |
|  |  |  |  |  |  |  | Prosthesis lifespan | Time to replacement and reason for replacement | Yes | No | No |
|  |  |  |  |  |  |  | Prosthetic aftercare | Number of aftercare procedures | Yes | No | No |
| Zuo and Wilkes 2016^41^ | Canada | Retrospective, observational, descriptive | Ad, Ch  Aur  Con, Onc, Tr, Inf, Bur, failed recon | Implant retained FP | N/A | 32.  RR = 21/29 (72%) | Satisfaction | Self-designed condition-specific questionnaire | No | N/A | No |
|  |  |  |  |  |  |  | Success | The ability to wear prosthesis as desired, independent of implant status | No | N/A | No |
| Kang et al, 2013^45^ | UK | Retrospective, observational, descriptive | Ad  Nas, Aur, Orb  Con, Onc, Tra, Inf, Bur | Implant retained FP | N/A | 6.  RR= 5/6 (83%) | Satisfaction | Self-designed condition-specific questionnaire | No | N/A | No |
| Kievit et al, 2013^39^ | The Netherlands | Retrospective  observational, descriptive | Ad, Ch  Aur  Con, Tra | Implant retained FP | N/A | 14.  RR = 10/14 (71%) | Quality of life (generic) | Short Form 12 (SF-12 Health Survey) | Yes | No | In other contexts |
|  |  |  |  |  |  |  | Quality of life (condition specific non-FP) | Glasgow Benefit Inventory (GBI) (used in otolaryngological interventions) | Yes | No | In other contexts |
|  |  |  |  |  |  |  |  | Rosenberg Self Esteem Scale (RSE) (used in plastic surgery procedures) | Yes | No | In other contexts |
|  |  |  |  |  |  |  | Satisfaction | Self-designed condition-specific questionnaire (additional questions) | No | N/A | No |
|  |  |  |  |  |  |  | Prosthesis lifespan | Time to replacement and reason for replacement | No | N/A | No |
| Curi et al, 2012^83^ | Brazil | Retrospective, observational, analytical | Ad, Ch  Nas, Aur, Orb, Com  Con, Onc, Tra | Implant retained FP, conventional manufacture | N/A | 56 participants | Prosthesis survival | Time to replacement | No | N/A | No |
| Si et al, 2012^57^ | China | Retrospective, observational, descriptive | Ad, Ch  Aur  Con, Tra, Bur, failed recon | Implant retained FP | N/A | 24.  RR not explicit | Satisfaction | Self-designed data collection (case note review) | No | N/A | No |
|  |  |  |  |  |  |  | Prosthesis lifespan | Time to replacement | No | N/A | No |
| Korus et al, 2011^38^ | Canada | Retrospective  observational, descriptive | Ad, Ch  Aur  Con, Onc, Tra, failed recon | Implant retained FP | N/A | 69. 63 sent surveys.  RR = 31/63 (49%) | Satisfaction | Self-designed condition-specific questionnaire | No | N/A | No |
| Ethunandan et al, 2010^84^ | UK | Retrospective, observational, descriptive | Ad  Nas  Onc | Implant retained FP | N/A | 34. | Prosthesis failure | Number of failures (FP that are not retained by implants) | No | N/A | No |
| Karakoca et al, 2010^73^ | Turkey | Retrospective, observational, descriptive | Ad, Ch  Nas, Aur, Orb  Con, Onc, Tra, Bur | Implant retained FP | N/A | 70. | Prosthesis survival | Time to replacement and reason for replacement | No | N/A | No |
|  |  |  |  |  |  |  | Clinician evaluation | Self-designed condition-specific data collection form | No | N/A | No |
|  |  |  |  |  |  |  | Complications | Numbers of technical complications |  |  |  |
| Karakoca Nemli et al, 2010^52^ | Turkey | Retrospective  observational, descriptive | Ad, Ch  Orb  Onc, Tra | Implant retained FP, conventional manufacture | N/A | 55 invited. 36 evaluated. RR not explicit. | Satisfaction | Condition-specific questionnaire based on other questionnaires by Hooper et al. 2005, Chang et al. 2005, Markt and Lemon 2001 | Yes | Yes - without explanation | No |
| Younis et al, 2010^37^ | UK | Retrospective, observational, descriptive | Ad, Ch  Aur  Con, Onc, Tra | Implant retained FP | N/A | 33.  RR = 20/33 (61%) | Satisfaction | Self-designed condition-specific questionnaire | No | N/A | No |
| Hamming et al, 2009^70^ | USA | Retrospective, observational, descriptive | Ch  Aur  Con | Implant retained FP | N/A | 8. | Daily duration of prosthesis wear | Self-designed data collection (case note review) | No | N/A | No |
|  |  |  |  |  |  |  | Complications | Numbers of biological complications |  |  |  |
| Sandner and Bloching, 2009^53^ | Germany | Retrospective, observational, analyticial | Ad  Nas, Com  Onc | Implant retained FP (some N/S) | Previous prosthesis, different retention | 11.  RR = 100% | Satisfaction | Condition-specific questionnaire proposed by Chang et al. 2005 | Yes | No | No |
| Visser et al, 2008^79^ | The Netherlands | Retrospective, observational, descriptive | Ad, Ch  Nas, Aur, Orb  Con, Onc, Tra | Implant retained FP | N/A | 95. | Prosthesis lifespan | Time to replacement and reason for replacement | No | N/A | No |
|  |  |  |  |  |  |  | Prosthetic aftercare | Number of aftercare procedures | No | N/A | No |
|  |  |  |  |  |  |  | Complications | Numbers of biological complications |  |  |  |
| Wagenblast et al, 2008^49^ | Germany | Retrospective, observational, descriptive | Ad  Nas, Aur  Onc | Implant retained FP | N/A | 5  RR = 100% | Satisfaction | Self-designed condition-specific questionnaire | No | N/A | No |
| Wright et al, 2008^78^ | USA | Retrospective, observational, descriptive | Ad, Ch  Aur  Con, Onc, Tra | Implant retained FP, conventional manufacture | N/A | 16 | Prosthesis success | Self-designed criteria for success assessing reversible and irreversible factors (including patient-reported and clinical factors) | No | N/A | No |
| Nassab et al, 2007^31^ | UK | Retrospective, observational, descriptive | Ad  Orb, Com  Onc | Mechanically retained FP or eye patch. | N/A | 32 (29 had FP).  RR = 100% | Satisfaction | Self-designed scale (good, fair, poor) | No | N/A | No |
| Honda et al, 2005^65^ | Japan | Retrospective, observational, descriptive | Ad  Nas, Aur, Orb  Onc, Tra | Implant retained FP | N/A | 12  RR= 8/12 (67%) | Psychological health | Cornell Medical Index Questionnaire (CMI) | Yes | No | No |
|  |  |  |  |  |  |  |  | Self-designed condition-specific questionnaire (psychological / satisfaction) | No | N/A | No |
|  |  |  |  |  |  |  | Complications | Numbers of technical complications |  |  |  |
| Rotenberg et al, 2002^10^ | Canada | Retrospective, observational, descriptive | Ch  Aur  Con, Tra | Implant retained FP, conventional manufacture. | N/A | 11.  7 (64%) met assessment criteria | Satisfaction | Condition-specific questionnaire adapted from an instrument by Anderson | No but refers to source | Yes - without explanation | No |
| Schoen et al, 2001^32^ | The Netherlands | Retrospective, observational, descriptive | Ad  Aur, Orb  Onc | Implant retained FP, conventional manufacture. | N/A | 26.  RR not explicit | Quality of life (generic) | Linear Analogue Self Assessment | Yes | No | No |
|  |  |  |  |  |  |  | Satisfaction | Self-designed condition-specific questionnaire | No | N/A | No |
|  |  |  |  |  |  |  |  | 10-point scale | No | N/A | No |
|  |  |  |  |  |  |  | Psychological health | Self-designed condition-specific questionnaire/scale | No | N/A | No |
|  |  |  |  |  |  |  | Functional comfort | Self-designed condition-specific scale | No | N/A | No |
| Westin et al, 1999^8^ | Sweden | Retrospective, observational, descriptive | Ad, Ch  Aur  Con, Onc, Tra, Bur | Implant retained FP | N/A | 99.  RR=85/92 (92%) | Quality of life (condition specific) | Condition-specific questionnaire based on questionnaire by Martin Deadman, Birmingham | No but refers to source | Yes - with explanation | No |
|  |  |  |  |  |  |  | Prosthetic aftercare | Clinical findings and aftercare procedures | No | N/A | No |
| Keerl et al, 1996^77^ | Germany | Retrospective  observational, descriptive | N/S  Nas, Aur, Orb  Con, Onc, Tra | Adhesive, implant or mechanically retained FP | N/A | 20.  RR = 100% | Appearance | Self-designed scale for grading of success by patient, surgeon and prosthetic designer | No | N/A | No |
| Berg et al, 1994^72^ | Sweden | Retrospective, observational, descriptive | Ad, Ch  Aur, Orb  Con, Onc, Tra | Implant retained FP | N/A | 22,  RR = 21 followed-up | Clinician evaluation | Self-designed condition-specific scale (may also have captured patient perspective) | No | N/A | No |
|  |  |  |  |  |  |  | Complications | Numbers of biological and technical complications |  |  |  |
| Granstrom et al, 1993^76^ | Sweden | Retrospective, observational, analytical | Ad, Ch  Aur  Con | Implant retained FP, conventional manufacture | Plastic surgery | 111 (62 FP, 37 plastic surgery) | Appearance | Self-designed scale indicating patient and surgeon satisfaction | No | N/A | No |
| Stevenson et al, 1993^46^ | UK | Retrospective, observational, descriptive | Ch  Aur  Con | Implant retained FP | N/A | 12 (9 had FP).  RR = 7/9 (78%) | Satisfaction | Self-designed condition-specific questionnaire | No | N/A | No |

| **Study** | **Setting** | **Design** | **Ages**  **Defect**  **Etiology** | **Intervention** | **Comparator** | **Participant numbers** | **Category** | **Measurement tool** | **Reference for tool** | **Tool modified** | **Validation reported** |
| --- | --- | --- | --- | --- | --- | --- | --- | --- | --- | --- | --- |
| Worrell et al, 2017^62^ | UK | Mixed methods | Ad  Nas, Orb, IO  Onc | Facial and/or oral prosthesis | N/A | 10 (6 had FP) | Quality of life (condition specific non FP) | University of Washington Quality of Life UW-QoL (used in head and neck cancer) (3 global quality of life questions added) | Yes | Yes | No |
| Roefs et al, 1984^71^ | The Netherlands | Mixed methods | Ad  Nas, Aur, Orb  N/S | FP | N/A | 14 | Clinician evaluation | Self-designed condition-specific scale to evaluate characteristics | No | N/A | No |

Supplemental Table 5. Study characteristics for mixed methods studies

Ad, adult > 18 years of age; Ch, child < 18 years of age; Aur, auricular; Nas, nasal; Orb, orbital; Ocu, ocular; Com, combined; IO, intraoral; Con, congenital; Onc, oncology; Tra, trauma; Inf, infection; Bur, burns; Exe, exenteration (reason not specified); FP, facial prosthesis; CAD-CAM, computer-aided design and computer-aided manufacturing; recon, reconstruction; RR, response rate; N/S, not specified; N/A, not applicable

Supplemental Table 6. Quality appraisal for each study using the QATSDD tool^16^

Please note that quality assessment tool selected that enabled evaluation of studies with diverse designs within single scale. This facilitated standardized approach across all study design groups and enabled broad quality comparisons to be drawn. It is acknowledged that QATSDD would not offer comprehensive assessment of every aspect of diverse range of evaluated study designs.

|  |  | Quality score for each quality criteria in the QATSDD tool | | | | | | | | | | | | | | | |  |  |
| --- | --- | --- | --- | --- | --- | --- | --- | --- | --- | --- | --- | --- | --- | --- | --- | --- | --- | --- | --- |
|  | Study type | Explicit theoretical framework | Statement of aims/ objectives in main body of report | Clear description of research setting | Evidence of sample size considered in terms of analysis | Representative sample of target group of a reasonable size | Description of procedure for data collection | Rationale for choice of data collection tool(s) | Detailed recruitment data | Statistical assessment of reliability and validity of measurement tool (Quantitative only) | Fit between stated research question and method of data collection (Quantitative only) | Fit between stated research question and format and content of data collection tool (Qualitative only) | Fit between research question and method of analysis | Good justification for analytic method selected | Assessment of reliability of analytical process (Qualitative only) | Evidence of user involvement in design | Strengths and limitations critically discussed | Total QATSDD score | Overall quality score as a percentage of the total possible score (%) |
| **Experimental studies (average rating 62%)** | | | | | | | | | | | | | | | | | | | |
| Abd El Salam et al, 2020 | Quantitative | 3 | 2 | 1 | 0 | 1 | 2 | 1 | 1 | 0 | 2 | N/A | 2 | 2 | N/A | 0 | 2 | 19 | 45 |
| Kiat-amnuay et al, 2010 and Anderson et al, 2013 | Quantitative | 3 | 2 | 3 | 2 | 2 | 3 | 1 | 3 | 2 | 3 | N/A | 3 | 3 | N/A | 1 | 2 | 33 | 79 |
| **Cross-sectional studies (average rating 45%)** | | | | | | | | | | | | | | | | | | | |
| de Oliveira et al, 2018 | Quantitative | 2 | 2 | 3 | 0 | 2 | 2 | 2 | 1 | 1 | 2 | N/A | 1 | 1 | N/A | 0 | 2 | 21 | 50 |
| Dings et al, 2018 | Quantitative | 2 | 2 | 2 | 1 | 2 | 2 | 1 | 3 | 2 | 2 | N/A | 2 | 1 | N/A | 0 | 1 | 23 | 55 |
| Becker et al, 2017 and Becker et al, 2016 | Quantitative | 1 | 1 | 1 | 0 | 2 | 1 | 2 | 1 | 1 | 2 | N/A | 2 | 0 | N/A | 1 | 2 | 17 | 40 |
| Kuiper et al, 2016 | Quantitative | 2 | 2 | 3 | 0 | 2 | 3 | 1 | 2 | 1 | 1 | N/A | 2 | 1 | N/A | 0 | 2 | 22 | 52 |
| Wondergem et al, 2016 | Quantitative | 2 | 1 | 1 | 0 | 2 | 1 | 1 | 2 | 0 | 2 | N/A | 2 | 0 | N/A | 0 | 1 | 15 | 36 |
| Smolarz-Wojnowska et al, 2014 | Quantitative | 1 | 2 | 1 | 0 | 2 | 1 | 0 | 2 | 0 | 2 | N/A | 1 | 0 | N/A | 0 | 1 | 13 | 31 |
| Atay et al, 2013 | Quantitative | 3 | 3 | 2 | 3 | 2 | 2 | 3 | 2 | 3 | 2 | N/A | 3 | 2 | N/A | 0 | 2 | 32 | 76 |
| Chang et al, 2005 | Quantitative | 3 | 3 | 1 | 1 | 2 | 3 | 2 | 1 | 0 | 2 | N/A | 2 | 2 | N/A | 0 | 2 | 24 | 57 |
| Hooper et al, 2005 | Quantitative | 2 | 2 | 1 | 0 | 2 | 2 | 1 | 2 | 0 | 2 | N/A | 2 | 0 | N/A | 2 | 0 | 18 | 43 |
| Horlock et al, 2005 | Quantitative | 2 | 2 | 1 | 0 | 2 | 2 | 2 | 2 | 0 | 2 | N/A | 2 | 1 | N/A | 0 | 2 | 20 | 48 |
| Markt and Lemon2001 | Quantitative | 2 | 2 | 3 | 0 | 2 | 3 | 2 | 2 | 0 | 2 | N/A | 2 | 0 | N/A | 0 | 1 | 21 | 50 |
| Jacobsson et al, 1992 | Quantitative | 1 | 2 | 1 | 0 | 2 | 1 | 1 | 3 | 0 | 1 | N/A | 1 | 0 | N/A | 0 | 0 | 13 | 31 |
| Chen et al, 1981 | Quantitative | 1 | 1 | 2 | 0 | 2 | 3 | 1 | 3 | 0 | 1 | N/A | 2 | 0 | N/A | 0 | 0 | 16 | 38 |
| Jebreil 1980 | Quantitative | 1 | 1 | 2 | 0 | 1 | 2 | 0 | 2 | 0 | 1 | N/A | 1 | 0 | N/A | 0 | 0 | 11 | 26 |
| **Prospective longitudinal observational studies (average rating 39%)** | | | | | | | | | | | | | | | | | | | |
| Faris et al, 2020 | Quantitative | 3 | 2 | 2 | 0 | 2 | 2 | 3 | 2 | 2 | 3 | N/A | 3 | 2 | N/A | 0 | 2 | 28 | 67 |
| Visser et al, 2020 | Quantitative | 2 | 2 | 3 | 0 | 2 | 3 | 1 | 3 | 0 | 2 | N/A | 1 | 0 | N/A | 0 | 2 | 21 | 50 |
| Zaoui et al, 2018 | Quantitative | 2 | 3 | 2 | 0 | 2 | 2 | 2 | 3 | 1 | 3 | N/A | 3 | 1 | N/A | 0 | 1 | 25 | 60 |
| Mevio et al, 2016 and Mevio et al, 2015 | Quantitative | 1 | 1 | 1 | 0 | 2 | 1 | 0 | 0 | 0 | 1 | N/A | 1 | 0 | N/A | 0 | 1 | 9 | 21 |
| Tam et al, 2014 | Quantitative | 2 | 2 | 3 | 1 | 1 | 2 | 2 | 2 | 1 | 2 | N/A | 2 | 2 | N/A | 0 | 2 | 24 | 57 |
| Nemli et al, 2013 | Quantitative | 2 | 2 | 2 | 1 | 2 | 2 | 2 | 3 | 1 | 2 | N/A | 2 | 1 | N/A | 2 | 2 | 26 | 62 |
| Aydin et al, 2008 | Quantitative | 0 | 2 | 0 | 1 | 1 | 3 | 0 | 0 | 0 | 2 | N/A | 1 | 1 | N/A | 0 | 0 | 11 | 26 |
| Arcuri et al, 1997 | Quantitative | 1 | 2 | 1 | 0 | 1 | 1 | 0 | 2 | 0 | 1 | N/A | 1 | 0 | N/A | 0 | 1 | 11 | 26 |
| Xu et al, 1997 | Quantitative | 0 | 1 | 0 | 0 | 2 | 1 | 0 | 0 | 0 | 0 | N/A | 0 | 1 | N/A | 0 | 0 | 5 | 12 |
| Tolman and Taylor 1996 and Tolman 1998 | Quantitative | 1 | 1 | 2 | 0 | 2 | 2 | 1 | 2 | 0 | 2 | N/A | 1 | 1 | N/A | 0 | 0 | 15 | 36 |
| Watson et al, 1995 | Quantitative | 1 | 0 | 1 | 0 | 2 | 2 | 2 | 1 | 0 | 0 | N/A | 0 | 1 | N/A | 0 | 0 | 10 | 24 |
| Reisberg and Lipner 1993 | Quantitative | 1 | 2 | 1 | 0 | 1 | 3 | 1 | 0 | 0 | 2 | N/A | 1 | 1 | N/A | 0 | 1 | 14 | 33 |
| Lowental and Sela 1982 | Quantitative | 1 | 2 | 1 | 0 | 2 | 3 | 2 | 2 | 0 | 2 | N/A | 2 | 1 | N/A | 0 | 0 | 18 | 43 |
| Sela and Lowental 1980 | Quantitative | 1 | 1 | 1 | 0 | 2 | 3 | 1 | 3 | 0 | 1 | N/A | 1 | 0 | N/A | 0 | 1 | 15 | 36 |
| **Retrospective longitudinal observational studies (average rating 42%)** | | | | | | | | | | | | | | | | | | | |
| Vijverberg et al, 2019 | Quantitative | 2 | 2 | 2 | 0 | 1 | 2 | 1 | 3 | 0 | 2 | N/A | 2 | 0 | N/A | 0 | 2 | 19 | 45 |
| Agarwal et al, 2018 | Quantitative | 2 | 2 | 2 | 0 | 2 | 2 | 0 | 3 | 0 | 2 | N/A | 2 | 1 | N/A | 0 | 1 | 19 | 45 |
| Bockey et al, 2018 | Quantitative | 1 | 2 | 1 | 0 | 2 | 2 | 2 | 2 | 2 | 2 | N/A | 2 | 1 | N/A | 0 | 2 | 21 | 50 |
| Papaspyrou et al, 2018 | Quantitative | 1 | 2 | 2 | 0 | 2 | 2 | 0 | 2 | 0 | 1 | N/A | 1 | 2 | N/A | 0 | 0 | 15 | 36 |
| Ryan et al, 2018 | Quantitative | 1 | 2 | 3 | 0 | 2 | 1 | 0 | 2 | 0 | 2 | N/A | 2 | 0 | N/A | 0 | 1 | 16 | 38 |
| Subramaniam et al, 2018 | Quantitative | 1 | 2 | 3 | 0 | 2 | 1 | 1 | 2 | 0 | 2 | N/A | 2 | 1 | N/A | 0 | 1 | 18 | 43 |
| Brandao et al, 2017 | Quantitative | 3 | 2 | 3 | 0 | 2 | 2 | 1 | 2 | 1 | 1 | N/A | 2 | 1 | N/A | 0 | 2 | 22 | 52 |
| DeSerres et al, 2017 | Quantitative | 3 | 2 | 3 | 0 | 2 | 2 | 2 | 3 | 0 | 2 | N/A | 3 | 1 | N/A | 0 | 2 | 25 | 60 |
| Korfage et al, 2016 | Quantitative | 3 | 2 | 3 | 0 | 2 | 2 | 2 | 2 | 0 | 2 | N/A | 2 | 1 | N/A | 0 | 1 | 22 | 52 |
| Zuo and Wilkes 2016 | Quantitative | 1 | 2 | 3 | 0 | 2 | 2 | 0 | 3 | 0 | 1 | N/A | 2 | 0 | N/A | 0 | 0 | 16 | 38 |
| Kang et al, 2013 | Quantitative | 2 | 1 | 1 | 0 | 1 | 1 | 0 | 1 | 0 | 2 | N/A | 1 | 0 | N/A | 0 | 2 | 12 | 29 |
| Kievit et al, 2013 | Quantitative | 3 | 2 | 2 | 0 | 1 | 3 | 3 | 3 | 1 | 3 | N/A | 2 | 2 | N/A | 0 | 1 | 26 | 62 |
| Curi et al, 2012 | Quantitative | 1 | 2 | 1 | 0 | 2 | 2 | 0 | 2 | 0 | 2 | N/A | 2 | 0 | N/A | 0 | 1 | 15 | 36 |
| Si et al, 2012 | Quantitative | 2 | 2 | 2 | 0 | 2 | 1 | 1 | 2 | 0 | 1 | N/A | 1 | 1 | N/A | 0 | 0 | 15 | 36 |
| Korus et al, 2011 | Quantitative | 3 | 2 | 3 | 0 | 2 | 3 | 1 | 2 | 0 | 2 | N/A | 2 | 1 | N/A | 0 | 1 | 22 | 52 |
| Ethunandan et al, 2010 | Quantitative | 3 | 2 | 2 | 0 | 2 | 1 | 0 | 2 | 0 | 2 | N/A | 2 | 0 | N/A | 0 | 1 | 17 | 40 |
| Karakoca et al, 2010 | Quantitative | 2 | 2 | 2 | 0 | 2 | 2 | 1 | 2 | 1 | 2 | N/A | 2 | 1 | N/A | 0 | 2 | 21 | 50 |
| Karakoca Nemli et al, 2010 | Quantitative | 2 | 2 | 2 | 0 | 2 | 3 | 2 | 3 | 1 | 2 | N/A | 2 | 2 | N/A | 0 | 2 | 25 | 60 |
| Younis et al, 2010 | Quantitative | 3 | 2 | 2 | 0 | 2 | 3 | 2 | 3 | 1 | 2 | N/A | 1 | 0 | N/A | 0 | 2 | 23 | 55 |
| Hamming et al, 2009 | Quantitative | 1 | 1 | 1 | 0 | 1 | 1 | 1 | 1 | 0 | 1 | N/A | 1 | 0 | N/A | 0 | 0 | 9 | 21 |
| Sandner and Bloching, 2009 | Quantitative | 2 | 2 | 2 | 0 | 1 | 2 | 2 | 2 | 0 | 1 | N/A | 1 | 0 | N/A | 0 | 1 | 16 | 38 |
| Visser et al, 2008 | Quantitative | 2 | 2 | 2 | 0 | 2 | 2 | 1 | 3 | 0 | 2 | N/A | 2 | 0 | N/A | 0 | 1 | 19 | 45 |
| Wagenblast et al, 2008 | Quantitative | 1 | 1 | 2 | 0 | 1 | 1 | 0 | 2 | 0 | 1 | N/A | 1 | 0 | N/A | 0 | 1 | 11 | 26 |
| Wright et al, 2008 | Quantitative | 2 | 2 | 2 | 0 | 2 | 2 | 2 | 2 | 0 | 2 | N/A | 1 | 0 | N/A | 0 | 1 | 18 | 43 |
| Nassab et al, 2007 | Quantitative | 1 | 1 | 1 | 0 | 2 | 2 | 0 | 1 | 0 | 1 | N/A | 1 | 0 | N/A | 0 | 0 | 10 | 24 |
| Honda et al, 2005 | Quantitative | 2 | 1 | 1 | 0 | 1 | 2 | 1 | 1 | 0 | 2 | N/A | 1 | 0 | N/A | 0 | 0 | 12 | 29 |
| Rotenberg et al, 2002 | Quantitative | 2 | 2 | 3 | 0 | 1 | 2 | 2 | 3 | 0 | 2 | N/A | 1 | 0 | N/A | 0 | 2 | 20 | 48 |
| Schoen et al, 2001 | Quantitative | 2 | 2 | 2 | 0 | 2 | 3 | 1 | 2 | 0 | 2 | N/A | 1 | 1 | N/A | 0 | 1 | 19 | 45 |
| Westin et al, 1999 | Quantitative | 1 | 2 | 2 | 0 | 3 | 3 | 1 | 2 | 0 | 2 | N/A | 2 | 2 | N/A | 0 | 2 | 22 | 52 |
| Keerl et al, 1996 | Quantitative | 0 | 1 | 2 | 0 | 2 | 2 | 0 | 1 | 0 | 1 | N/A | 1 | 0 | N/A | 0 | 0 | 10 | 24 |
| Berg et al, 1994 | Quantitative | 1 | 1 | 1 | 0 | 1 | 1 | 0 | 2 | 0 | 1 | N/A | 1 | 0 | N/A | 0 | 1 | 10 | 24 |
| Granstrom et al, 1993 | Quantitative | 1 | 1 | 2 | 0 | 2 | 1 | 1 | 1 | 0 | 2 | N/A | 1 | 1 | N/A | 0 | 1 | 14 | 33 |
| Stevenson et al, 1993 | Quantitative | 2 | 1 | 3 | 0 | 1 | 2 | 1 | 3 | 1 | 2 | N/A | 1 | 0 | N/A | 0 | 1 | 18 | 43 |
| **Mixed methods studies (average rating = 38%)** | | | | | | | | | | | | | | | | | | | |
| Worrell et al, 2017 | Mixed methods | 2 | 2 | 1 | 1 | 2 | 1 | 1 | 2 | 0 | 2 | 1 | 2 | 1 | 0 | 0 | 1 | 19 | 40 |
| Roefs et al, 1984 | Mixed methods | 2 | 1 | 1 | 0 | 1 | 2 | 1 | 2 | 1 | 1 | 2 | 1 | 0 | 1 | 0 | 1 | 17 | 35 |
